# Predicting spatial spread of rabies in skunk populations using surveillance data reported by the public

**DOI:** 10.1371/journal.pntd.0005822

**Published:** 2017-07-31

**Authors:** Kim M. Pepin, Amy J. Davis, Daniel G. Streicker, Justin W. Fischer, Kurt C. VerCauteren, Amy T. Gilbert

**Affiliations:** 1 National Wildlife Research Center, United States Department of Agriculture, Wildlife Services, Fort Collins, Colorado, United States of America; 2 Institute of Biodiversity, Animal Health and Comparative Medicine, University of Glasgow, Glasgow, Scotland; 3 Medical Research Council-University of Glasgow Centre for Virus Research, Glasgow, Scotland; Universidad Nacional Mayor de San Marcos, PERU

## Abstract

**Background:**

Prevention and control of wildlife disease invasions relies on the ability to predict spatio-temporal dynamics and understand the role of factors driving spread rates, such as seasonality and transmission distance. Passive disease surveillance (i.e., case reports by public) is a common method of monitoring emergence of wildlife diseases, but can be challenging to interpret due to spatial biases and limitations in data quantity and quality.

**Methodology/Principal findings:**

We obtained passive rabies surveillance data from dead striped skunks (*Mephitis mephitis*) in an epizootic in northern Colorado, USA. We developed a dynamic patch-occupancy model which predicts spatio-temporal spreading while accounting for heterogeneous sampling. We estimated the distance travelled per transmission event, direction of invasion, rate of spatial spread, and effects of infection density and season. We also estimated mean transmission distance and rates of spatial spread using a phylogeographic approach on a subsample of viral sequences from the same epizootic. Both the occupancy and phylogeographic approaches predicted similar rates of spatio-temporal spread. Estimated mean transmission distances were 2.3 km (95% Highest Posterior Density (HPD_95_): 0.02, 11.9; phylogeographic) and 3.9 km (95% credible intervals (CI_95_): 1.4, 11.3; occupancy). Estimated rates of spatial spread in km/year were: 29.8 (HPD_95_: 20.8, 39.8; phylogeographic, branch velocity, homogenous model), 22.6 (HPD_95_: 15.3, 29.7; phylogeographic, diffusion rate, homogenous model) and 21.1 (CI_95_: 16.7, 25.5; occupancy). Initial colonization probability was twice as high in spring relative to fall.

**Conclusions/Significance:**

Skunk-to-skunk transmission was primarily local (< 4 km) suggesting that if interventions were needed, they could be applied at the wave front. Slower viral invasions of skunk rabies in western USA compared to a similar epizootic in raccoons in the eastern USA implies host species or landscape factors underlie the dynamics of rabies invasions. Our framework provides a straightforward method for estimating rates of spatial spread of wildlife diseases.

## Author summary

Rabies is a deadly zoonotic infection with a global distribution. In 2012, an epizootic of skunk rabies established in northern Colorado, USA and spread rapidly through three counties. The epizootic was documented through reports of dead skunks by the public. We examined the reports to determine how rapidly rabies was moving and which factors could explain the patterns of spread. We compared these estimates of spatial movement of rabies to those obtained from analyzing rabies genetic sequences that we obtained from some of the dead skunks reported by the public. By both methods, we found the virus was moving south at a little over 20 km/year and that most transmission between skunks occurred at short distances (< 4 km). Rabies was most likely to spread to new areas during the first half of the year, when skunk populations were producing new offspring. Our genetic model suggested that roads and rivers in the study landscape did not affect movement speed of rabies. We developed a framework that used the spatial data in the public reports to predict where and when skunk rabies would occur next. This framework could be used on public health surveillance data for other diseases or countries.

## Introduction

A central focus for disease ecologists and epidemiologists is to quantify processes that determine geographic spread of disease [1]. Surveillance systems, which rely on reporting by the public [2–5], provide data that can improve understanding disease dynamics and planning interventions. However, passive surveillance data are challenging to interpret because the underlying sampling design is opportunistic. Raw patterns may depend on observation processes that produce a biased representation of disease occurrence. Interpretation of passive surveillance data from wildlife populations can be especially challenging because the underlying ecological processes, such as host population density, distribution, and demographic dynamics, are often unknown [e.g., 6]. In these cases a phenomenological method that does not rely on explicit representation of often unavailable host ecological data, may be valuable for quantifying disease spread—especially when surveillance data are too sparse, or disease prevalence too low, to enable estimation of multiple unknown parameters representing non-linear processes from both the host and disease dynamics.

Rabies virus (RABV) is a globally-distributed zoonotic pathogen that circulates naturally in a variety of carnivore and bat host species and has among the highest case fatality rate of known infectious diseases [7]. The principal burden of human and animal cases is associated with domestic dog populations [8], but emergence in wild carnivores has been observed, especially in areas where domestic dog rabies has been managed or controlled [9,10], or in areas with a long history of disease absence [11]. Risk of rabies transmission from wildlife is traditionally monitored through public health surveillance systems, which involve voluntary reports by the public of domestic animal or human exposures to potentially sick wildlife (appearance of atypical behavior, and especially signs of neurologic illness), followed by diagnostic testing [7]. Animal movement can have significant consequences on geographic spread of RABV [12], but the transmission distance during individual infections, an important component of geographic spreading, is poorly documented. Analysis of spatial public-health surveillance data have the potential to improve our understanding of the geographic spreading processes of RABVs, which will in turn help with planning prevention and response strategies, and prioritizing resources in space and time.

In the United States of America (USA), rabies infections of humans and animals became nationally reportable during 1938 [13]. There are several distinct enzootic lineages of RABVs circulating in bats and wild carnivores in the USA [14], though most control and management efforts are focused on raccoon RABV [15]. The South Central Skunk (SCSK) variant of RABV was likely first detected in Texas as early as 1953 [16], although typing methods which could detect and identify this particular variant were not reported until 1986 [17]. Recent studies have documented that this variant of RABV has been expanding in geographic distribution [18] and recently invaded novel areas in the USA, causing epizootics in the northern part of Colorado for the first time [19]. This well-documented invasion presents an opportunity to quantify spatial emergence dynamics of skunk RABVs, and assess the validity of estimating spatial epidemiological parameters from surveillance data reported by the public.

Ecologists have developed occupancy models for quantifying species invasion processes [20], which are essentially the same phenomena as the emergence and geographic spread of novel pathogens. Occupancy is the process of a target species or pathogen being present or absent across space. Dynamic occupancy processes additionally consider a time component. Occupancy frameworks can incorporate an ecological process(es) of geographic spread and observation error separately, allowing for clearer interpretation of the influence of factors driving each process. Recently, occupancy models have begun to be applied to disease systems for estimating pathogen prevalence [21–23] but are under-utilized for quantifying parameters that describe the spatial spread of disease.

Using surveillance data from the SCSK epizootic in Colorado, we developed a dynamic patch-occupancy model to jointly estimate parameters describing the spatial spread of rabies while accounting for variable sampling effort. Considering surveillance data from dead skunks, we used the framework to: 1) quantify the rate of geographic spread and transmission distance per infection; 2) quantify effects of local infection density, seasonality and direction on the probability of geographic invasion; and 3) predict the occupancy probability and prevalence of RABV on the landscape through space and time. We also conducted a phylogeographic analysis of viral genetic data from a subset of the reported rabies positive skunks to validate the estimated rates of spatial spread and transmission distance that we estimated using the occupancy model. The occupancy framework we present is simple to implement and can be used for inferring rates and direction of geographic spread in a variety of emerging disease systems, providing basic insight for understanding disease emergence and practical insight for risk assessment and response planning.

## Methods

### Ethics statement

The decision and implementation of euthanasia of animals was conducted by county authorities, before samples were received for the current study (i.e., we had no role in this). For the current study, the State of Colorado Department of Natural Resources issued annual Scientific Collection Licenses (14SALV2060, 15SALV2060) in order for us to receive a subset of dead carcasses for testing.

### Surveillance data

**Fig 1:**
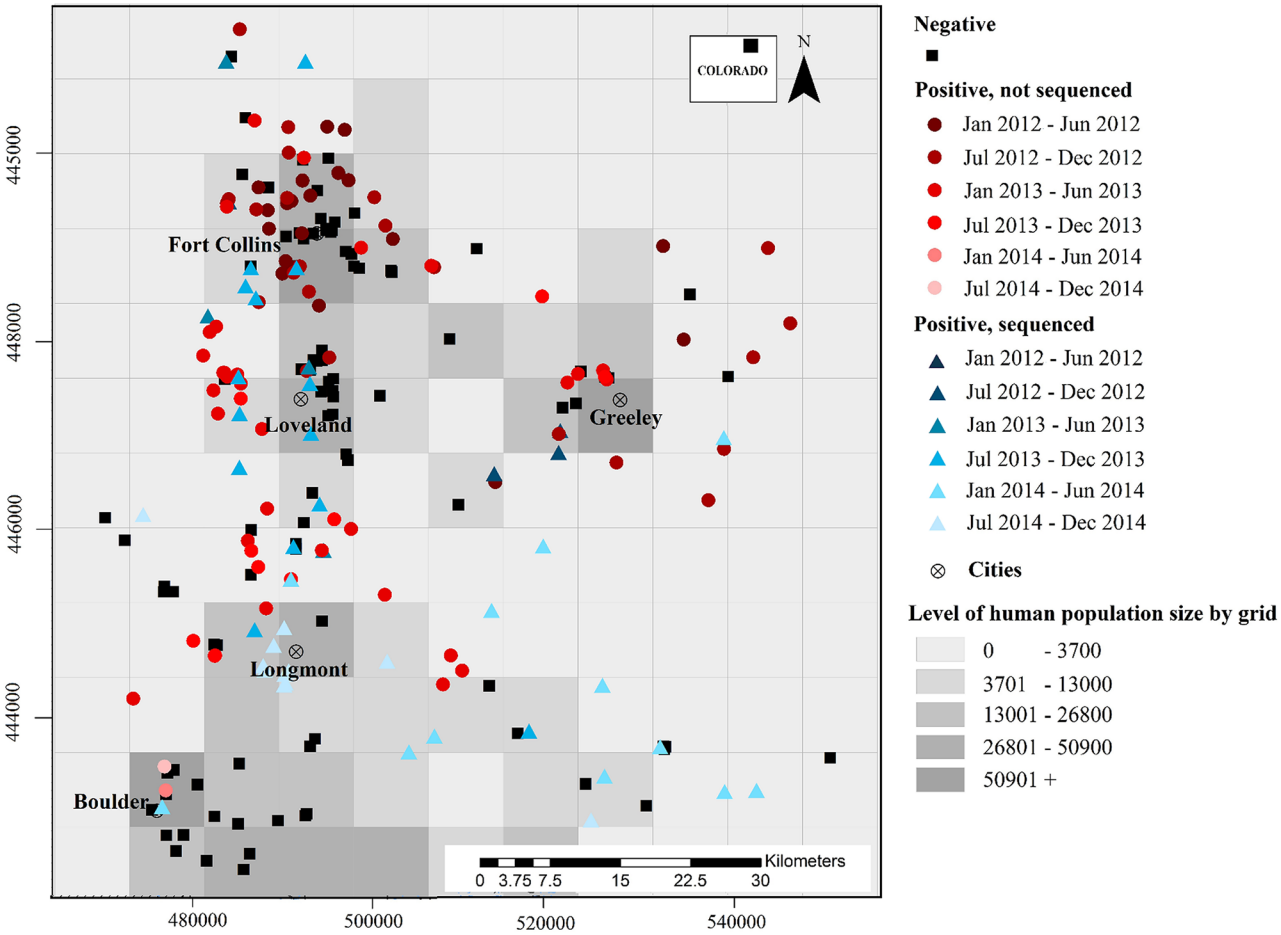
Map of study area. Each dot represents the location where a dead skunk was reported and subsequently tested for rabies. Grid cells show the scale at which the study area was gridded (8 x 8 km sites, i = 1, …, M).

The study area included 8448 km^2^ (88 x 96 km; Fig 1) of Larimer, Boulder and Weld counties, Colorado, USA. Skunk samples were obtained by reports made by public to local health departments, which decided whether to submit a skunk for rabies testing to one of two diagnostic laboratories in the state. The decision and implementation of euthanasia of animals was conducted by county authorities, before samples were received for the current study. For the current study, the State of Colorado Department of Natural Resources issued annual Scientific Collection Licenses (14SALV2060, 15SALV2060) in order for us to receive a subset of dead carcasses for testing. There was no vaccination campaign (trap-vaccinate-release or oral rabies vaccination) targeting wildlife during the epizootic, but there were alerts through local public media and leash laws in effect.

Strange-acting or dead mammals found on the landscape with no reported human or domestic animal contact were not typically tested by the local health departments—especially later in the study period when the perceived risk of rabies infection was much lower and funding for testing had decreased. Because of the ongoing epizootic, these samples were referred for testing by United States Department of Agriculture, Animal and Plant Health Inspection Service, Wildlife Services, National Wildlife Research Center (NWRC); hereafter referred to as ‘enhanced surveillance’. Enhanced surveillance accounted for 0%, 1% and 47% of surveillance data in 2012, 2013 and 2014 respectively (Table SM1.1 in S1 Text). Enhanced surveillance samples were identified through the public health surveillance system by the local health departments, but with carcass referral to NWRC for rabies testing. Carcasses were referred to NWRC when the perceived risk to public health was low (i.e., due to a lack of contact with humans or pets). Because the rabies epizootic was waning in 2014, the high proportion of enhanced surveillance in 2014 relative to 2012 and 2013 did not contribute much extra information (except for improving uncertainty levels) using the occupancy model. Thus, typical surveillance systems could likely make the same type of inferences, but would show greater uncertainty as sampling decreased. An address matching-geocoding technique, using ArcGIS 10.3 (Environmental Systems Research Institute, Redlands, CA, USA), was used to convert street addresses of the case reports to UTM (Universal Transverse Mercator) coordinates.

Before 2012, carnivore rabies had not been detected in the study area for several decades. From 2012–2014 there were 246 skunk reports and 379 non-skunk terrestrial animal reports tested for rabies (Fig 1, Table SM1.2 in S1 Text), with a total of 139 rabies-positive skunks (raw prevalence ~ 57%, Table SM1.2 in S1 Text), and 21 rabies-positive cases from the 379 non-skunk terrestrial species tested (5.5% positive; Table SM1.2 in S1 Text) including: raccoons (6), bison (2), foxes (6) cats (2), horses (2), cows (2), coyotes (1). All non-skunk terrestrial animals were excluded from the analysis because they comprised such a small proportion of the samples and because surveillance efforts may have differed in some of these hosts—especially raccoons which are higher density and more peridomestic, and were experiencing an ongoing epizootic of canine distemper virus. Human population data from 2013 [24] were organized at the grid-cell scale (8 x 8km) to be included in the occupancy model as a potential factor contributing to reporting rate.

### Occupancy model formulation

We used a discrete-time, dynamic, patch-occupancy model to quantify the occurrence probability of skunk rabies in space and time. In a typical occupancy framework, there is the partially observable occupancy process and an observation process that is conditional on the occupancy process. For this dynamic model the occupancy process describes the probability of rabies being present by site (grid cells) and time (months). The ecological process of rabies occurrence changed over grid cells and months due to the local component processes of colonization, local extinction, and persistence. Within the latent ecological process, we estimated important parameters describing the initial colonization dynamics (i.e., spatial invasion) of skunk rabies, specifically: direction of invasion, distance to nearest infected grid cell, and density of infection in the local neighborhood. Conditional on the latent ecological process of occupancy, we modeled the observation process as the probability that an animal sampled has rabies, given that rabies is present (prevalence given rabies occupancy). Within the observation process, we considered the effects of human population size on the proportion of skunks that were rabies positive. Thus, our approach allowed us to quantify parameters underlying the spatio-temporal dynamics of geographic spread while accounting for imperfect detection. For the occupancy model, we assumed the landscape was homogenous because our genetic model suggested that spread rates were homogenous across the landscape and we did not have enough degrees of freedom to incorporate more parameters in our occupancy model.

We used a monthly time step to scale with the incubation period for rabies virus in skunks [25,26]. We divided the study area (much of Larimer, Weld and Boulder Counties in Northern Colorado, USA) into 8 x 8 km grid cells (“patches” in occupancy modeling terms; Fig 1). This spatial scale was much larger than a skunk home range size [27,28] suggesting that local transmission (i.e., due to skunk movements alone) should be primarily within grid cells or nearest-neighbor grid cells. Also, at this spatial scale, there were multiple samples collected within grids (Fig 1) at a given time step (mean number of samples in grids with samples: 3.6 sampled skunks, range 1–19 sampled skunks), which is necessary for estimating grid-cell level prevalence and informing the observation process, but at finer spatial scales multiple samples per grid cell and time step were rare. Thus, because of the sparse sampling intensity in our study area, the 8 x 8 km grid cell size was the smallest size we could choose for approximating the skunk home range (~ 2 x 2 km; [25, 26]) and hence skunk-to-skunk transmission distances.

Data on the presence of rabies in dead skunks (sampling unit = 1 skunk) were collected repeatedly in grid cells i = 1, …, M during each month t = 1, …, T (spanning January 2012 through December 2014). We modeled the true occupancy status *z*_*i*, *t*_ conditioned on the previous time step *z*_*i*, *t-1*_, as a latent Bernoulli variable defined by parameter *Ψ*_*i*, *t*_ (occupancy probability), where *z*_*i*, *t*_ = 0 indicates that grid cell *i* is not occupied (no rabies in skunks) in time step *t*, and *z*_*i*, *t*_ = 1 indicates that grid cell *i* is occupied (at least one skunk with rabies) in time step *t*.

$$z_{i,t}|z_{i,t-1}\sim Bern\left(\Psi_{i,t}\right)$$ (1)

We assumed the initial occupancy state for each grid cell (*z*_*i*, *1*_) was also a Bernoulli random variable described by occupancy probability *Ψ*_*i*, *1*_, which had a Uniform prior distribution (*Ψ*_*i*, *1*_~Unif(0, 1)). We allowed occupancy probability in grid cell *i* at time *t* (*Ψ*_*i*, *t*_) to be determined by three types of local dynamic processes: initial colonization (γ_i,t_), recolonization (ζ_i,t_) and persistence (ϕ_i,t_), which were conditional on the latent occupancy status in the previous time step (*z*_*i*, *t-1*_).

$$\Psi_{i,t}=\gamma_{i,t-1}\left(1-z_{i,t-1}\right)\left(1-A_{i,t-1}\right)+\zeta_{i,t-1}\left(1-z_{i,t-1}\right)A_{i,t-1}+\phi_{i,t-1}z_{i,t-1}$$ (2)

We used parameter *A*_*i*, *t*_ to distinguish an initial colonization event from subsequent colonization events. Although it is more typical to consider only occupancy and extinction dynamics in occupancy models, we further distinguished initial colonization from recolonization (as in [29]) because we were most interested in factors (described below) driving spatial invasion into new sites. We quantified the effects of multiple different factors (direction, distance to nearest infected neighbor, neighborhood infection density (Eqs 3–8) on initial colonization probability (γ_i,t-1_), and treated the other processes as inherent contributors to occupancy probability (*Ψ*_i,t_) without quantifying their potential drivers (i.e. we used a global parameter for persistence- *ϕ*_*it*_ ~ *Beta*(*α*_*ϕ*_, *β*_*ϕ*_) and recolonization—*ζ*_*it*_ ~ *Beta*(*α*_*ζ*_, *β*_*ζ*_). For γ_i,t-1_, an intercept term (β_o_) absorbed non-spatial effects (Eq 3). Direction (north-south (*N*) or east-west (*E*)) was modeled as increasing integers (1 representing the southernmost grids and 12 representing the northernmost grids for N; and 1 representing the westernmost grids and 11 representing the easternmost grids for E; Eq 4, β_k_, where k = 1, …, K for the number of initial colonization effects considered). East-west direction was represented similarly but using columns in the grid. We modeled an interaction between direction and a trend in time (*T*) by multiplying the direction covariate data by the time step (*N*_*i*_ · *T* or *E*_*i*_ · *T*). We calculated distance to the nearest infected grid cell by taking the minimum distance for all pairwise distances between the centroid of target grid cell i* in time *t* and all other grid cell centroids (*i*) in time *t-1* (d_ii*_). We estimated the relationship of initial colonization probability and distance using an exponential decay function which included a parameter describing the decay rate of initial colonization probability (α) with distance and a scaling parameter relative to maximum initial colonization probability (β_k_, Eq 5). We estimated the local neighborhood infection density for grid cell i* in time *t* using the occupancy prevalence in all immediate grid-cell neighbors (queen’s neighbors; *j* = 1, …, J; note *j*’s are a special subset of *i*—restricted to the closest neighbors of i*) in time step *t-1* and estimated its effect with parameter β_k_ (Eq 6). We incorporated a seasonal effect as a factor where one level represented spring/summer (Feb.–Aug.) and the second level represented fall/winter (Sept.–Jan.) (Eq 7). We chose these time frames because the literature and surveillance data suggest peaks of rabies in spring and summer following arousal and mating activities, and subsequent peaks in fall and winter associated with dispersal and contact involving susceptible young of the year [16, 25]. We modelled the infection density (Eq 6) and distance effects (Eq 5) separately because we were interested in the separate effects of distance versus infection intensity. Together these effects describe the spatial kernel for local transmission. Additionally, we modelled a weighted spatial kernel (Eq 8) which accounted for the density of neighbors with infections but discounted the impact an infected cells at further distances from the grid of interest (d_ii*_ represents the distance between grid cell ‘*i*' and the grid cell of interest ‘i*’).

*logit(γ*_*i*,*t-1*_*)* =

$$\begin{array}{cc} \beta_{0} & \left(\text{non spatial}\right) \end{array}$$ (3)

$$\begin{array}{cc} \beta_{1}N+\beta_{2}T+\beta_{3}N*T & \left(\text{direction by time}\right) \end{array}$$ (4)

$$\begin{array}{cc} \beta_{4}e^{-\alpha min(d_{{}_{ii*}}}{}^{)} & \left(\text{transmission distance}\right) \end{array}$$ (5)

$$\begin{array}{cc} \beta_{5}\left(1/J\right)\sum^{J}{}_{j=1}\left[z_{j,t-1}\right] & \left(\text{local neighborhood effect}\right) \end{array}$$ (6)

$$\begin{array}{cc} \beta_{6}S & \left(\text{season – Feb. – Aug. vs Sept. – Jan.}\right) \end{array}$$ (7)

$$\begin{array}{cc} \beta_{7}\sum_{zi,t-1=1}\left[1/d_{ii*}\right] & \left(\text{weighted spatial kernel}\right) \end{array}$$ (8)

We considered these effects (Eqs 4–8) separately and additively (up to three effects including the Eq 3) in our model selection procedure (described below). We did not present the fullest model because parameter estimates became inconsistent when more than three additional effects were in the model. Persistence and recolonization were important processes in the occupancy dynamics, thus we modeled them explicitly using Beta prior distributions with shape and scale parameters (*α*_*ϕ*_ = 1, *β*_*ϕ*_ = 1, *α*_*ζ*_ = 1, *β*_*ζ*_ = 1). We did not include covariates that could potentially drive persistence and recolonization because we were only interested in the process of spatial spread (i.e., initial colonization). Prior distributions for all β_k_ parameters were: β_k_ ~ Norm(0, 1), except for the scaling parameter for the exponential decay model (Eq 5) which was modeled as a Gamma(5, 1).

For the observation layer of our model, we represented the number of skunks that were positive for rabies, *y*, in grid cell *i* at time *t* when rabies was present as the observed data using a binomial distribution where *p* was the estimated prevalence of rabies in dead skunks (given occupancy) and the number of trials (*R*_*i*, *t*_) were the observed number of samples collected in grid cell *i* at time *t*.

$$y_{i,t}=\left\{0,for\;z_{i,t}=0,i=1,\ldots ,M;Bin\left(p,R_{i,t}\right),for\;z_{i,t}=1,t=1,\ldots ,T\right\}$$ (9)

To account for variation in rabies detection due to variation in human population size, we examined prevalence as a linear function of human population size (*N*).

$$logit\left(p_{i}\right)=\eta_{0}+\eta_{1}log_{10}\left(N_{i}\right)$$ (10)

We only investigated this effect in the intercept-only model (Eq 3) and the two-effect model we were most interested in (Table SM2.1 in S1 Text for list of all models that were fit). The general model specification is given in section SM2.1 in S1 Text.

To calculate the posterior distribution for the parameters of interest, we fit models using a Markov chain Monte Carlo (MCMC) algorithm with a Gibbs sampler including Metropolis-Hastings steps [30] custom written in program R [31]. Posterior estimates for the data are based on 50,000 iterations of the MCMC algorithm with the first 5,000 iterations discarded as burn-in. Convergence and mixing were assessed graphically. Example MCMC chains and posterior distributions are given in SM2.1 in S1 Text. Convergence of the best predictive model was additionally assessed using the Gelman-Rubin statistic [30]. The joint and conditional distributions are specified in SM2.3 in S1 Text. We evaluated the ability of our models to recover parameters by simulating data from known parameter values and estimating the parameter values using our fitting framework (Model Validation Method in SM5 in S1 Text).

### Model selection and evaluation

We used a combination of Watanabe Akaike Information Criterion (WAIC) [32, 33], Area Under the receiver operator Curve (AUC) [34], and leave-one-out cross validation (looCV) [31] to compare the importance of different effects on *γ* and *p* and assess goodness-of-fit (presented in Table 1, schematic of work flow shown in Fig. SM3.1). WAIC is a model selection criterion based on the posterior predictive distribution, and was used to compare fits of models. WAIC was not used to compare models with human population modelled on prevalence (*p*) because the data were different and thus the WAIC values would not be comparable. AUC is a measure of how well variation is explained by the model—in our case, the ability to distinguish a presence from an absence—and was used to assess how well a particular model explained the data (i.e. a measure of goodness of fit as in [35]). LooCV is a measure of the model’s ability to predict out of sample, and thus was used to compare predictive ability among models. When comparing predictive ability between models we used both AUC and looCV.

**Table 1:** Model selection and fit statistics for within- and out-of-sample predictions.

#	Effects on initial colonization probability (γ)	*p*	Within sample			Out of sample		
			WAIC	AUC1	AUC2	AUC1	AUC2	looCV
1	·	-	0.804	0.95	0.64	0.78	0.62	-205.71
1b	·	Pop	NA	1	0.64	0.81	0.62	-218.22
2	Distance	-	0.803	0.96	0.7	0.78	0.69	-205.72
3	NxT	-	0.803	0.96	0.66	0.78	0.66	-205.72
4	ExT	-	0.804	0.95	0.66	0.78	0.67	-205.72
5	Neighborhood	-	0.803	0.96	0.72	0.78	0.7	-205.71
6	Season	-	0.802	0.95	0.71	0.78	0.7	-205.72
7	Kernel	-	0.804	0.96	0.72	0.78	0.7	-205.72
8	Distance + NxT	-	0.804	0.96	0.68	0.78	0.7	-205.95
9	Distance + Neighborhood	-	0.804	0.96	0.76	0.78	0.74	-205.99
10	NxT + Neighborhood	-	0.804	0.96	0.71	0.78	0.69	-205.79
11	Distance + Season	-	0.804	0.96	0.76	0.78	0.75	-205.77
12	NxT + Season	-	0.804	0.96	0.66	0.78	0.71	-205.84
13	Neighborhood + Season	-	0.804	0.95	0.73	0.78	0.71	-205.72
14	Kernel + N*T	-	0.804	0.96	0.71	0.78	0.69	-205.79
15	Kernel + Season	-	0.804	0.97	0.75	0.78	0.68	-205.99
16*	Neighborhood + Season + Distance	-	0.804	0.96	0.76	0.78	0.73	-205.88
9b	Distance + Neighborhood	Pop	NA	0.99	0.75	0.8	0.74	-218.7
11b**	Distance + Season	Pop	NA	0.99	0.78	0.81	0.72	-219.71
16b	Neighborhood + Season + Distance	Pop	NA	0.99	0.74	0.8	0.72	-218.05

**Fig 2:**
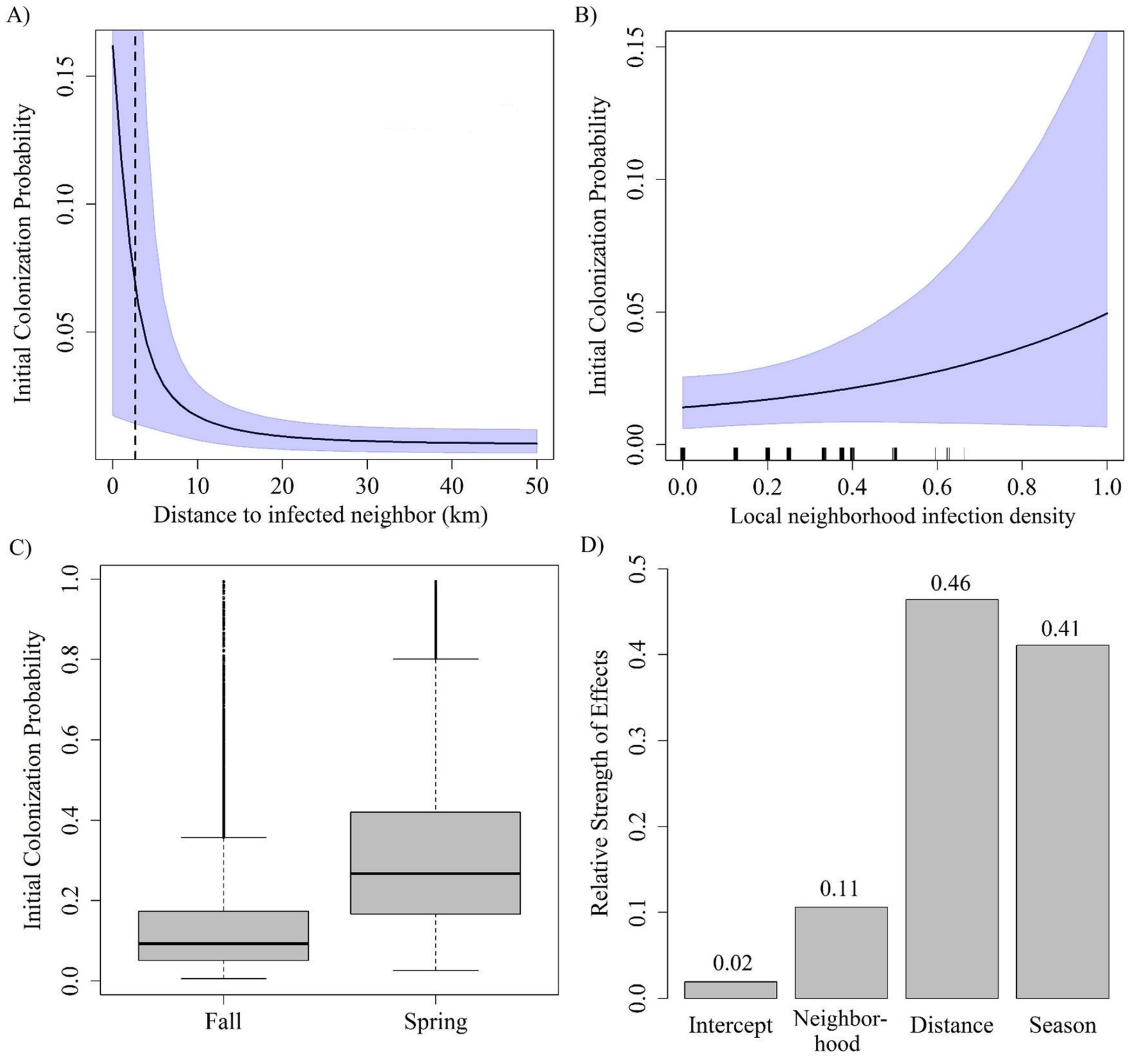
Effects of covariates on initial colonization probability. Predicted γ as a function of different factors using Model 16 Transmission distance = 3.9 km 95% CI (1.4, 11.3) (neighborhood+distance+season, Table 1). A) Decay of initial colonization probability with distance to nearest infected grid cell in km. B) Proportion of local neighborhood (“queen’s neighbors”) infected. C) Season factor with two levels. Median values of initial colonization probability are indicated by the horizontal black line. D) Relative contribution of different effects (see section SM5 in S1 Text for calculations). Shading in A and B indicates 95% credible intervals.

*Model used to estimate effects in Fig 2;

**Best predictive model, used to make predictions in Figs 4 and 5; · = intercept only, - = no effect, Pop = log human population size effect on prevalence parameter *p*, Distance = effect of distance to nearest infection, Neighborhood = local neighborhood infection density effect, N∙T = north-south by time directional effect, Season = factor with two levels (spring/summer: Feb.-Aug. and Fall/Winter: Sept.-Jan.), E∙T = east-west by time directional effect, -WAIC = Watanabe Akaike Information Criteria (section SM4.1 in S1 Text), AUC1 = Area Under the Curve for observed positive counts (section SM4.2 in S1 Text), AUC2 = Area Under the Curve for latent occupancy process (section SM4.3 in S1 Text), looCV = leave-one-out cross validation score (section SM4.4 in S1 Text), NA = not available; we did not display these WAIC values because they cannot be compared to the WAIC values from the models without Pop.

We calculated WAIC and AUC (Section SM4 in S1 Text) for fits to the full data as well as the looCV predictions. WAIC is preferable to DIC (Deviance Information Criterion—another Bayesian method for model selection) for model selection using hierarchical models [32] because it considers the posterior predictive distribution explicitly and penalizes for complexity of model structure, not just the number of parameters. Similar to DIC, lower values of WAIC indicate a better model of the data. However, in contrast to DIC [36], there is no standard quantitative difference between WAIC values from alternative models that indicates a significant difference between them (i.e., the only criterion is that lower is better). We presented two AUC scores: 1) AUC1 measured predictive ability of *p* using predicted *y*’s from posterior values of *z* and observed *y*’s, 2) AUC2 measured predictive ability of Ψ using the posterior values of *z* and the observed *y*’s transformed to binary data. For the out-of-sample predictions, we conducted looCV for each point in the data (using all other points as the training data) and presented means of AUC1 and AUC2 as measures of predictive ability.

### Out-of-sample prediction

To further evaluate the predictive ability of our approach we predicted occupancy status over space and time using only parameters estimated from the “best predictive model” (i.e., considering out-of-sample statistics for AUC1, AUC2 and looCV) and the sample size data. First, we fit the best model to the full data and then predicted all *y*_*i*, *t*_ from the posterior predictive distribution [30]. Each predicted *y*_*i*, *t*_ depended on the predicted *y*_*i*, *t-1*_, rather than the data, but the actual data for sample size (*R*_*i*, *t*_) were used in the prediction in order to scale *y*_*i*, *t*_ predictions appropriately.

### Rates of spatial spread from occupancy model

Using the model with the best out-of-sample looCV score, we predicted grid cell occupancy over time (*z*_*i*, *t*_; rabies presence -1, or absence -0), and estimated the rate of southerly spread of the predictions using regression. First, we obtained the *z*_*i*, *t*_ values from each MCMC iteration (minus the burn in) for grids across time. Each grid cell corresponded to a distance in kilometers from the southernmost border of the study area (e.g., 1, …, 88 km; “North value”). For *z*_*i*, *t*_ = 1 values, we ran a simple linear regression where the independent values were the months in time and the dependent values were the north values. The slope of this regression represents the monthly rate of southerly spread. We then converted this to an annual rate of southerly spread by multiplying the monthly rate by 12. We calculated the annual variance using the delta method [37].

### Genetic sequencing

To verify spatial patterns indicated by our occupancy model, we sequenced the whole or partial glycoprotein (G, 1575 base pairs [bp]) and the non-coding region between the glycoprotein and polymerase genes (GL, 560 bp) for 53 viruses collected in Colorado between August 2012 and December 2014 (Table SM1.3 in S1 Text). We added sequence data from 20 viruses collected through May 2015 (27.4% of the total genetic dataset) to increase the precision of parameters of the molecular evolutionary models. We confirmed that these additional sequences comprised an extension of the same epizootic though preliminary phylogenetic analysis and the absence of changes in the inferred rate of spread in 2015 which could have biased our overall spread rate. Rabies virus RNA was extracted from brainstem tissue samples of rabies positive skunks using Trizol reagent following the manufacturer’s protocol. The conversion of RNA to cDNA (RT) and primary PCR amplification was accomplished using a Superscript III One-step RT-PCR system with Platinum Taq DNA Polymerase (Invitrogen), targeting a 2,135bp region including the full glycoprotein gene with previously published primers [18,38]. PCR products were visualized by UV-light on a 2% agarose gel with ethidium bromide, and cleaned using ExoSap-IT (Affymetrix) following the manufacturer’s protocol. Sequencing reactions were performed using Big Dye Terminator v.3.1 (Applied Biosystems), with flanking and internal primers as previously described [18,38]. Sequencing products were cleaned using Sephadex G-50 columns (GE Healthcare) and run on a 3130 or 3500 analyzer (Applied Biosystems). Forward and reverse sequences were aligned in Sequencher v.5.2.4 (Gene Codes Corporation), and ambiguities were resolved visually. Alignment of full or partial glycoprotein sequences was performed using BioEdit v.7.2.0.

### Phylogeographic analysis of viral spread

Continuous phylogeographic analysis was conducted in BEAST v. 1.8.4 [39]. Briefly, this method estimates the phylogenetic history connecting samples and conducts ancestral state reconstruction of the latitudes and longitudes of inferred nodes using time and location-annotated sequence data. Phylogenetic uncertainty is accommodated by summarizing estimates across the posterior distribution of trees from a Bayesian search. Analyses in TempEst (software that analyzes correlations between the temporal and genetic distances between sequences) showed evidence of a molecular clock signal in the coding (G) and non-coding (GL) regions of the RABV genome, but faster evolution in the G-L region compared to G [40]. Our BEAST analysis therefore modeled a single tree topology (given that the samples represent the same underlying epidemic history) using different molecular clock and substitution models to allow for differences in evolutionary rates in different parts of the RABV genome. Preliminary BEAST runs using the lognormal relaxed molecular clock indicated little variation in rates among branches, indicating the use of the strict molecular clock for both partitions, which was supported by equivocal differences in Bayes Factors (BF) between models assuming strict or relaxed molecular clocks (BF = 1.5 in favor of strict clocks) [41]. Final runs used customized substitution models for the following data partitions: G codon positions 1+2 = TPM1uf; G codon position 3 = TIM1+G, GL = TPM1uf+I, as suggested by AIC in jModeltest2 [42]. Among the 3 random walk phylogeographic models tested (homogenous, Cauchy and gamma), marginal likelihoods estimated by the stepping stone method were highest in the homogenous model (-3574.86) followed by the gamma (-3577.27) and Cauchy (-3594.05) models, suggesting relatively little variation in spread rates among branches or little power to detect such variation. To account for the possibility of among branch variation, we present the results of the gamma model, but note that parameter estimates were nearly identical in the similarly supported homogeneous rate model. All analyses used the Bayesian skyline model of demographic growth. MCMC chains were run for 100 million generations which generated effective samples sizes >200 after removal of burn-in.

We used the Seraphim package of R to calculate viral diffusion rates from 500 randomly selected (post burn-in) trees from the posterior distribution of the BEAST analysis [43]. We estimated viral spread rates as (i) the “mean branch velocity” calculated as the average velocity across the branches of each tree, averaged across all trees and (ii) the weighted “diffusion rate”, obtained by summarizing distances and times spanning each tree and taking the average of that value across all trees (i.e., sum distances/sum time lengths over entire tree). To calculate the transmission distance per viral generation (i.e., per infected animal), we divided the distance traversed along each branch by the expected number of infections along that branch, assuming a generation time of 30 days, which corresponds to the incubation period of rabies virus in skunks [25,26]. However, the substitution rates that we estimated (G: 4.1x10^-4^ substitutions per site per year (95% highest posterior density (HPD) = 2.3–6.05x10^-4^); GL: 8.7x10^-4^ [HPD_95_ = 4.1–13.4 x10^-4^]), imply that some transmission events would be expected to occur without detectable evolution in the partial genomes that we sequenced. This led to branch lengths that were less than the assumed generation time of RABV, which caused an upward bias in inferred transmission distances per infection. We corrected for this bias by forcing all branches to have a minimum generation time of 1 infection.

## Results

### Model selection

The probability of initial colonization (γ) was best explained by the minimum distance to infections, infection density in the local neighborhood, season, and spatial kernel (see AUC2 of Models 1–7; Table 1). Similarly, the best two-effect models included pairs of these effects (distance + neighborhood, distance + season, kernel + season; Models 9, 11, 15; Table 1). Accounting for direction (i.e., N∙T or E∙T) provided more minor improvements to quantifying initial colonization probability (AUC2 Table 1). The inclusion of human population size on *p* (rabies prevalence in occupied grid cells) significantly improved predictive power of the model (see AUC1 and looCV in Table 1, Models 1b, 9b, 11b, 16b) because it explained some of the prevalence variation due to differences in the number of samples reported by humans. We considered Model 11b to be the best predictive model because it had the lowest looCV, and we used this model for prediction. However, in order to study the effects of the most significant single predictors (neighborhood, season and distance), we fit Model 16 to quantify the relative importance of these factors (results presented in Fig 2).

### Effects on initial colonization probability

**Fig 3:**
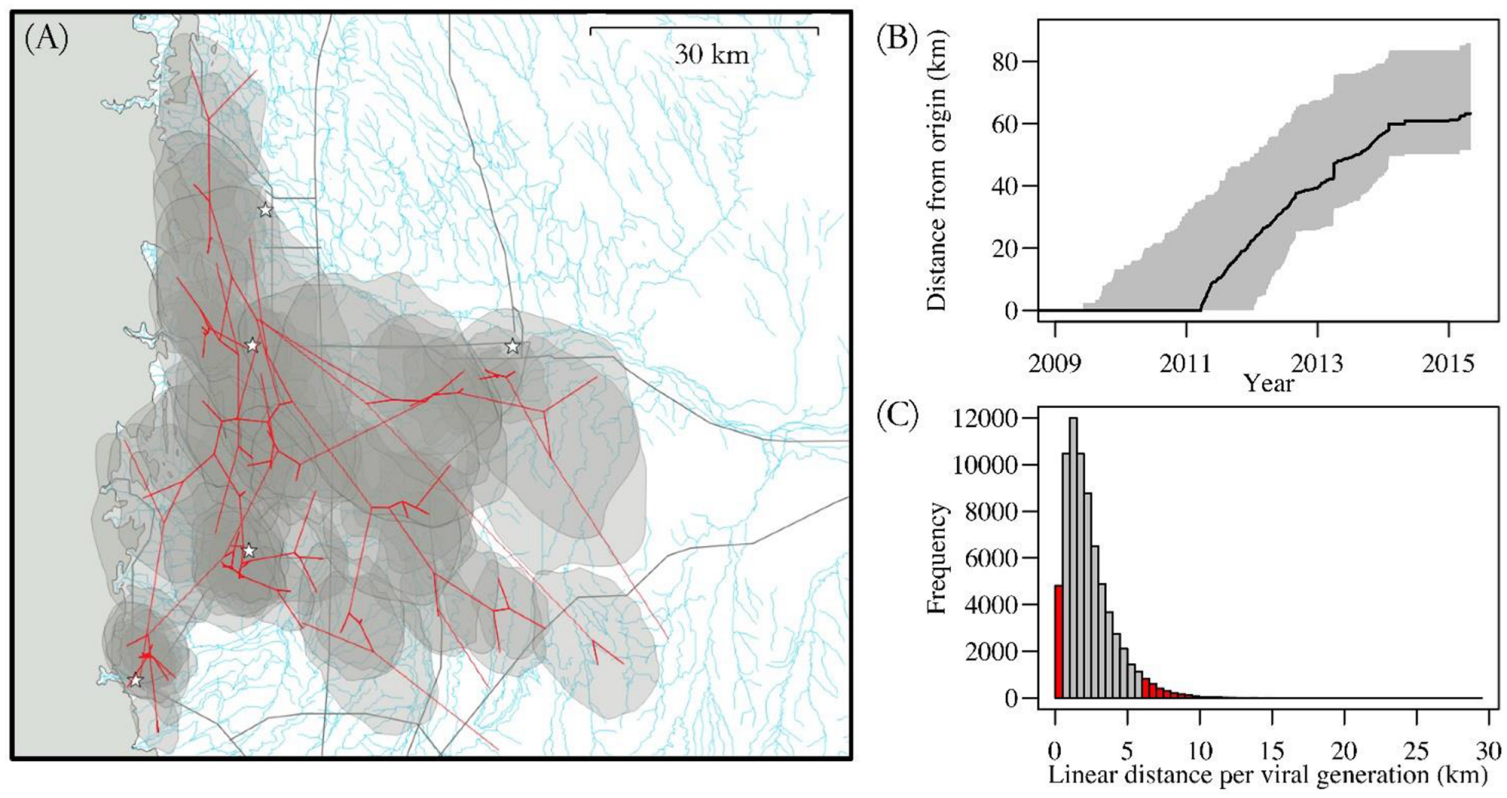
Phylogeographic inference of viral spatial dynamics. A) Spatio-temporal projection of the maximum clade credibility tree from the phylogeographic analysis. Transparent polygons indicate the 80% HPD of infected area through time. Grey shading on the left indicates landscape that is above 1829 m. Grey lines indicate major highways. Blue lines indicate all waterways, including very minor and ephemeral ones. Stars indicate the centers of major cities (as in Fig 1). B) Spatial expansion of RABV in skunks, displayed as the distance from the inferred epizootic origin in the phylogeographic analysis. Grey shading indicates the HPD_95_. The solid black line is the median distance. C) The distance traversed by each branch in the phylogenetic tree divided by the expected number of infections along that branch, assuming a generation time of 30 days. The histogram shows distances calculated from the 144 branches of 500 randomly-selected trees from the posterior distribution of the phylogeographic analysis. Grey bars are the inner HPD_95_.

We modeled the effect of distance to nearest infection using an exponential decay function. Using the estimated decay rate parameter, we calculated the distance at which initial colonization probability decayed to half its maximum (referred to as “transmission distance”) using the asymptotic limit as the minimum (see section SM54 in S1 Text for calculation). We interpreted the transmission distance as a measure of skunk-to-skunk transmission distance. Transmission distance was 3.9 km (95% credible intervals (CI_95_): 1.4, 11.3; Fig 2A). Similarly, using the genetic data independently, the mean distance per viral generation (another proxy for skunk-to-skunk transmission distance) was estimated to be 2.3 km (95% Highest Posterior Density (HPD_95_) = 0.04–5.7; Fig 3C).

Initial colonization probability increased exponentially with local neighborhood infection density, especially after 0.2 (i.e., > 1grid cell occupied; Fig 2B, Model 16, Table 1). Initial colonization probability was substantially higher during the spring/summer season relative to the fall/winter season (Fig 2C, Model 16). Of the 76% of variation in occupancy probability that was explained by the three-factor model (AUC2, Model 16, Table 1), 57% of initial colonization probability was explained by local effects (distance or neighborhood), 41% was explained by season and 2% was explained by other factors (which could include translocation or other unknown factors; Fig 2D). Although direction explained less variation in initial occupancy probability relative to other factors (Table 1, Models 3 and 4 versus Models 2, 5, 6, and 7), there appeared to be a significant increase in initial colonization probability in the southerly direction with time during the study period indicating southerly spread of the disease (Fig SR1.1 in S1 Figures). However, east-west movement over time did not show significant visual trends (Fig SR1.2 in S1 Figures).

### Prediction

**Fig 4:**
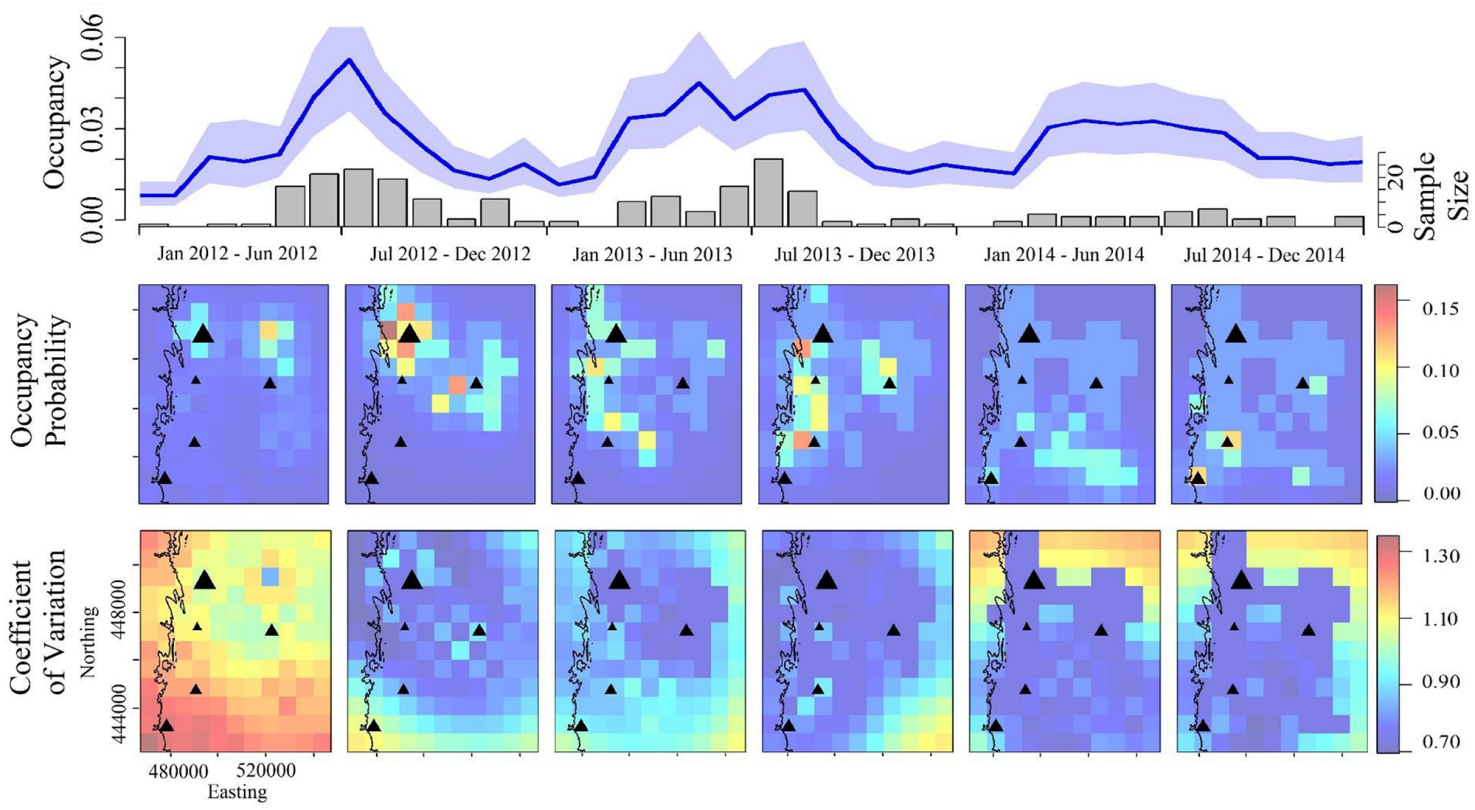
Rabies occupancy probability over time. Top. Occupancy probability for all sites over time (blue line, shading: 95% credible intervals) using Model 11b, Table 1). Grey bars: number of samples collected. Middle. Occupancy probability in space and time. Bottom. Coefficient of variation for occupancy probabilities over space and time. Middle and bottom: Data were aggregated over a 6-month time frame (corresponding to the X-axis labels of the top row plot). Black triangles represent city locations (Fort Collins, Greeley, Boulder and Longmont); sizes scaled to the human population size of the cities. Topographical divide from Fig 1, which indicates land above 1829 m (6000 feet), is indicated by the black line.

**Fig 5:**
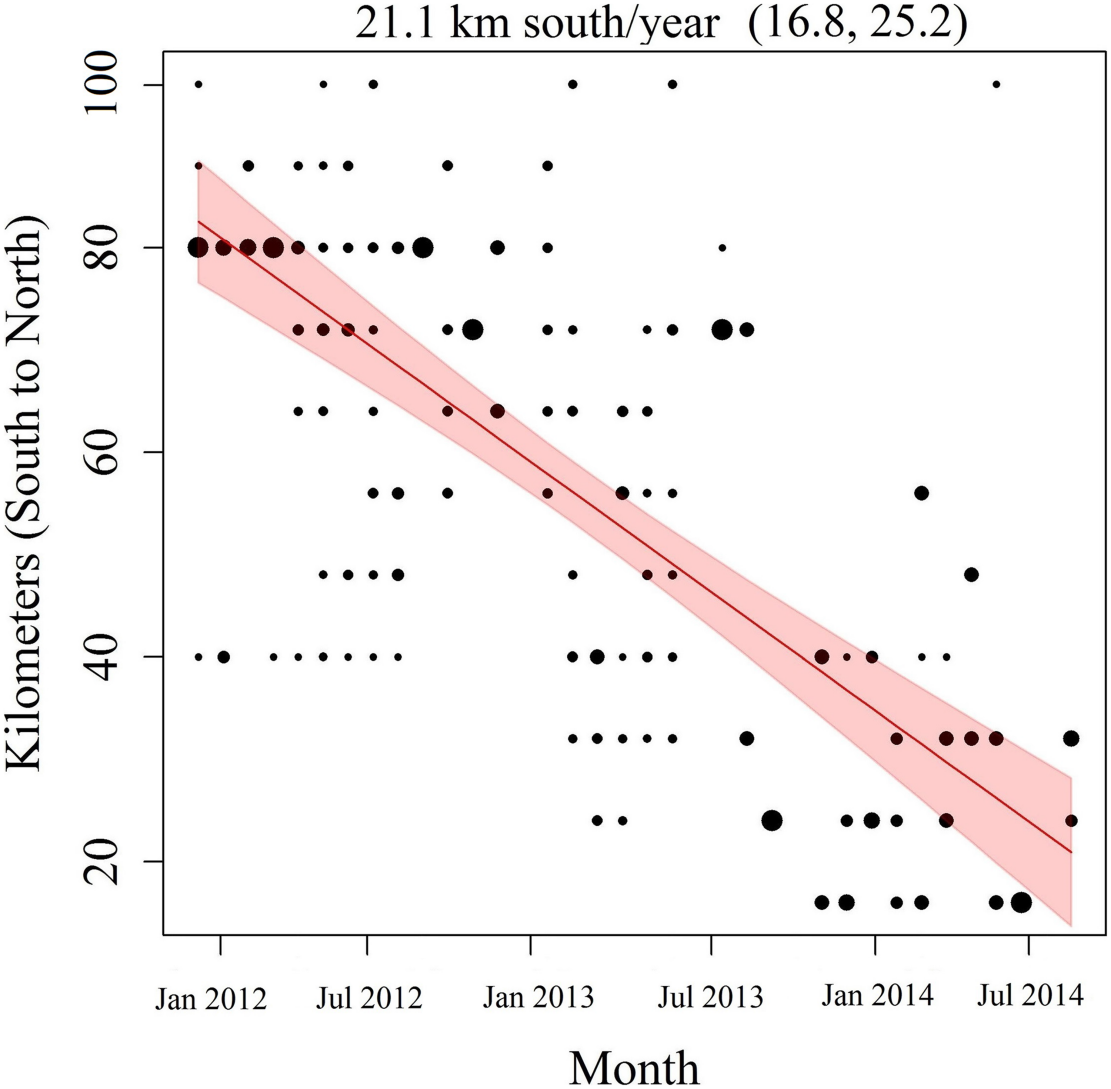
Rates of spatial spread. Model 11b was used to predict spatial spread of rabies. Points are the predicted distance in km of new colonizations from the southern-most grid-cell row. Size of the points corresponds to numbers of new colonizations at a given distance from the southern-most row. The slope (red line) gives a monthly rate of southerly spread (21.1 km/yr). Shaded area gives the 95% confidence intervals of the slope. The 6-month intervals shown on the X-axis correspond to those in Fig 4.

The “best” predictive model we examined (Model 11b, Table 1) performed very well at predicting rabies cases in space and time using parameters trained on different data than the data being predicted (Table 1, out-of-sample prediction AUC1 & AUC2). The model also performed very well at out-of-sample prediction using only the initial conditions, sample size data and parameters fit to the full data (Fig SR2.1 in S1 Figures). Average occupancy probability across the study area was low over time (Fig 4, top row, mean: 0.034, CI_95_: 0.006–0.24) but prevalence in sampled dead skunks in occupied grid cells was high (Table 2, mean: 0.91, CI_95_: 0.86, 0.95). Occupancy probability was predicted to decrease substantially in the north and increase in the south during the time course of the study period (Fig 4, Figs SR3.1 & SR3.2 in S1 Figures). Also, earlier during the time course occupied patches were larger and more contiguous whereas later occupied patches became more fragmented and isolated (Fig 4). The overall rate of southerly spread estimated by the occupancy model was predicted to be 21.1 km/year (CI_95_: 16.7, 25.5, Fig 5). This was similar to the phylogeographic diffusion rate assuming either gamma 21.8 [HPD_95_: 14.9–29.0] or homogenous rate 22.6 [HPD_95_: 15.3–29.7] models. Mean branch velocities from the phylogeographic models were slightly higher but had overlapping HPD_95_s with the genetic and occupancy-based models (gamma: 28.4 [HPD_95_: 19.6–39.8] or homogenous 29.8 [HPD_95_: 20.8–39.8]).

**Table 2:** Parameter estimates from the best predictive model of occupancy probability (Model 11b, Table 1) and the phylogeographic model.

	Parameter Estimates (uncertainty)
**Occupancy probability**	0.034 (0.006, 0.236)
**Persistence probability**	0.22 (0.15, 0.31)
**Initial colonization probability**	0.018 (0.01, 0.06)
**Re-colonization probability**	0.032 (0.02, 0.04)
**Prevalence**	0.91 (0.86, 0.95)
**AUC1**	0.96
**AUC2**	0.76
**Local neighborhood parameter (β**_**k**_**)**	1.15 (-0.53, 2.86)
**Increase in γ for 1 additional infected neighbor**^a^	0.002 (-0.000, 0.016)
**Mean transmission distance in km**^a^ **(occupancy)**	3.9 (1.4, 11.3)
**Mean transmission distance in km (phylogeographic)**	2.3 (0.02, 5.7)
**Rate of southerly spread in km/year (occupancy)**	21.1 (16.7, 25.5)
**Mean branch velocity (phylogeographic)**	28.4 (19.6–39.8) (gamma) 29.8 (20.8–39.8) (homog)
**Diffusion rate (phylogeographic)**	21.8 (14.9–29.0) (gamma) 22.6 (15.3–29.7) (homog)
**Total number of positives**	140

Rate of southerly spread = slope of relationship between kilometers moved south and time Fig 5); Numbers in brackets (uncertainty) indicate 95% credible intervals for occupancy results and 95% highest posterior density for phylogegraphic results;

^**a**^See section SM5 in S1 Text for derivation of these values; Gamma and homogenous (homog) refer to different assumptions about the level of branch heterogeneity in the phylogeographic models. Models using Cauchy distributed rates were less supported (BF = 16.8 and 19.2 in favor of homogeneous and gamma, respectively) and are omitted from the table.

## Discussion

We used an occupancy modeling framework to understand and predict the spatial spread dynamics of an important zoonotic disease in a reservoir host species. The framework produced good out-of-sample predictions of spatial dynamics despite our relatively small dataset, suggesting it can be useful for predicting spread in other surveillance systems that rely on passive surveillance data. We converted occupancy probabilities into rates of directional spread, which is useful for planning interventions and allocating resources for disease management. The rates of spatial spread predicted by the occupancy model appeared similar to those predicted by phylogeographic analyses. Thus, our phenomenological approach for estimating rates of spatial spread may provide accurate prediction of spatial spread rates based on parameters estimated in the recent past for a particular region. While the phylogeographic method incorporates additional information regarding connectivity between cases (i.e., genetic sequence similarity), the occupancy model produces similar results with only the spatial and temporal information. The phylogeographic method has the advantage of not requiring negative samples, while the occupancy approach has the advantage of not requiring genetic data. Each may therefore be suited to address alternative data gaps typical to surveillance systems. Both the phylogeographic and occupancy approaches can be sensitive to sampling intensity and scale, such that interpretation of results should consider sampling design.

Our approaches also present an alternative to detailed mechanistic models of disease transmission which may require more data than are available, such as knowledge of host population sizes and connectivity (e.g., [44]) (although we did not have host demographic data to explicitly test for differences between our phenomenological approach and a more mechanistic approach). The occupancy framework is flexible for testing the role of different contact heterogeneities. Any desired connectivity pattern could be incorporated by modifying the spatial terms using another description of spatio-temporal connectivity between rabies-positive cells. Similarly, if geographic barriers are important [45], or if there are data on ecological processes of known importance such as host density, additional terms could be added to occupancy processes to quantify these effects and improve prediction. In addition, some surveillance systems (e.g., the National Rabies Management Program of the United States Department of Agriculture, Animal and Plant Health Inspection Service, Wildlife Services) are comprised of multiple types of surveillance data (e.g., passive methods such as: public health reports, road kill reports, landowner reports of nuisance animals, or active methods such as: trapping), which each introduce their own sampling biases. The occupancy framework is flexible for incorporating multiple methods of surveillance simultaneously and, in the observation model, allowing for quantification of effects due to the type of surveillance data, which can in turn inform the design of surveillance. In situations where vaccination or other interventions are applied, effects of the intervention could be quantified by incorporating those data as a covariate in the latent ecological process. Thus, our framework could be used to identify the magnitude of intervention effects and facilitate planning of where additional interventions are needed.

The directional rates we estimated by both the occupancy and phylogeographic models were twice as high as those estimated for SCSK RABV by [18] using a phylogeographic approach (10 km/year; range 4–12 km) on data from south-central USA. One reason for this discrepancy could be that the analysis of [18] averaged rates over a long time period and large geographic area (multiple US states), whereas our study covered a short time period within three counties in a single state. From a level IV ecoregion perspective, our study site included only 4 distinct ecoregions (Rolling sand plains, Flat to rolling plains, Front range fans and Foothill shrublands), while that of [18] included 50–100 ecoregion types. Thus, the landscape within our study area was less heterogeneous than the landscape within the study of [18], which could partly explain differences in rates of spatial spread of rabies. Also, estimates from longer time scales may have included re-emergence dynamics in addition to initial invasion dynamics (reducing inferred rates of spatial spread). However, the rates we estimated are significantly lower than those found during the initial emergence of RABV in raccoons in the eastern USA (~38 km/year) [43,46], suggesting that skunk ecology, at least in south and western USA, leads to slower geographic spread of RABV. This is consistent with a previous study which used landscape resistance models and showed that skunks and raccoons used different movement corridors on the landscape [47]. Also, skunk rabies variants are 8.1 times less likely to be transmitted to foxes relative to raccoon rabies variants [14]–their lower propensity to spillover into longer-ranging hosts could contribute to slower spatial spreading.

Our mean estimates of individual-level transmission distance (~ 3.9 km by the occupancy model and 2.3 km by the phylogeographic model) were higher than the mean transmission distance estimated for dogs in the Serengeti, Tanzania (0.88 km) [48]. This estimate, which was based on infection reports, suggest a much closer mean transmission distance than was estimated in study of canine rabies in South Africa which was conducted using genetic data [mean distance between the most probable linked cases = 14.9 km; [49]. The latter study suggests a significant influence of anthropogenic movement of dogs, which is unlikely for skunks, and no rabies-positive dogs were reported. Although there were some canine species (6 foxes and 1 coyote) found to be positive for SCSK in our study area, these reports only comprised 5% of the rabies-positive reports suggesting that these longer-ranging hosts may only have made minor contributions to spatial spreading. Thus, our estimates of mean transmission distance likely reflect distances at which skunks move naturally during the contact and transmission process. Our approach required that multiple samples were collected in the same grid cell at the same time step in at least some of the grid cell/time steps (here we had a total of 180 grid cells with > 1 sample in the same time step over a total of 132 grid cells x 36 time steps = 4752 (3.8%)). Based on this requirement, we used a larger spatial scale (8 x 8 km) than skunk home range size (~ 2 x 2 km, would have been preferable). Thus, our estimates of transmission distance were likely biased high. For example, the transmission distance estimated in the occupancy model (3.9 km) was about half the width of a gird cell. As we used grid-cell centroids to calculate distances between cells, our approach may not have allowed for smaller resolution of transmission distance (relative to the phylogenetic model) even though the rate of spread on the landscape was slower in the occupancy model. Although local transmission explained the majority of the occupancy probability, seasonality effects were also substantial (41% of explained variation). We found initial colonization probability to be approximately twice as high in the spring/summer months relative to the fall winter months, which is consistent with other studies of rabies seasonal prevalence in skunks [16].

Some previous studies of spatial spread of RABV in raccoons showed that landscape barriers such as major rivers, highways or mountains influence the direction and speed of spread [45,50], while other studies have not observed a strong effect of rivers and major roads [47,50]. In certain landscapes, the presence of suitable host habitat was observed to influence spread [51] and presence [52] of skunk RABV. Another study on spatial spread of skunk RABV in the mid-western USA observed differential impacts of river barriers depending on the skunk RABV variant [53]. Most of our study area was comprised of relatively flat terrain east of the Rocky Mountains. There were only two samples on the west side in the Rocky Mountains (none above 2509 meters), and our study area did not include the other side of the Rocky mountains, thus our models were unable to test effects of high-altitude barriers explicitly. In addition, there were no major rivers (e.g., Colorado River) in our study area, but there were two moderately sizes rivers (Poudre River and Big Thompson River) and one major road (Interstate 25). Although the rivers and roads could have been easily incorporated into our occupancy framework, we did not include them because: 1) the phylogenetic analysis suggested minimal heterogeneity in rates of spatial spread across the rivers and roads in our study area, and 2) our dataset was small relative to the number of parameters we wanted to estimate (i.e., we prioritized quantification of spatial parameters). Also, maps of the genetic data showed closely related rabies variants on both sides of the most major road (Interstate 25) and rivers (Poudre River, Big Thompson River) throughout the study. Thus, although we did not explicitly test for effects of rivers and roads with our occupancy model, plots of the genetic data and the phylogenetic analyses suggested that the rivers and roads in our study area may not have presented major barriers that could reinforce controls against geographic spread [54]. If an intervention were deemed to be a necessary and/or cost-effective response, the primarily local transmission suggested that potential interventions could be applied as a relatively thin barrier (10–20 km) at the wave front to curb invasion, which is less than half the ~40km width typically used during aerial vaccination operation against raccoon rabies in the USA (Rich Chipman, National Rabies Management Program, personal communication). The strong seasonal difference in initial colonization probability suggests that interventions in the spring may be more effective than interventions in the fall.

Our model predicted that geographic spread tended to be mostly local and north-south, resulting in a wave-like pattern of geographic invasion. The phylogeographic analyses suggested that the invading viruses were new to Northern Colorado, such that the skunk population in Northern Colorado had not seen this virus in the recent past and was likely to be highly susceptible to invasion. We hypothesize that the reason we found a stronger tendency for movement of rabies from north to south instead of east to west, may be due to the underlying distribution of the skunk population. In our study area (called the Colorado Front Range), the human population forms a north-south belt, sandwiched by lower density regions to the west (in the mountains) and the east (in the plains). Although the literature does not suggest a consistent relationship of increasing skunk density across the rural to urban gradient [55, 56], it is possible that skunk abundance was higher running north-south than east-west, which could partly explain our directional effects.

Forecasting spatio-temporal disease spread in any system is challenging, but especially so for wildlife diseases due to an often poor understanding of host population abundance and ecology. Occupancy models present an alternative to approaches that rely on host demographic data, while quantifying rates and patterns of geographic spread based on underlying ecological processes. Although we accounted for observation error (by modeling the effect of human population size on prevalence), we did not have detailed data informing the observation process. For example, the study area included many natural areas spanning large regions where there are no human residents, but potentially heavy public usage, year-round. Accounting for reporting bias through collection of system-specific appropriate metadata (e.g. temporal usage patterns of the landscape by humans, geographic differences in the likelihood of human reporting) could improve geographic spread predictions using occupancy modeling. Our study showed how quantifying spatial parameters governing geographic spread can provide important ecological insights for understanding spatial epidemiology of rabies, conducting risk assessment and planning interventions—contributing to the toolbox of approaches for guiding management of disease invasions [57].

## Supporting information

S1 Text Methods. Additional tables describing surveillance data, a schematic describing the modeling strategy, and additional description of model formulation, validation, selection, and goodness of fit methodology.
(PDF)

S1 Figures Results. Additional figures showing parameter fits and spatial spread.
(PDF)

S1 Data (CSV)
