# Supplementary material for: Predicting spatial spread of rabies in skunk populations using surveillance data reported by the public
Source: PLoS Negl Trop Dis. 2017 Jul 31;11(7):e0005822. doi: 10.1371/journal.pntd.0005822 (PMC5552346; doi:10.1371/journal.pntd.0005822)
Supplement: S1 Text — Additional tables describing surveillance data, a schematic describing the modeling strategy, and additional description of model formulation, validation, selection, and goodness of fit methodology. (PDF) [file pntd.0005822.s001.pdf]

# Supporting Information 1: Methods "Predicting spatial spread of rabies in wildlife populations using surveillance data reported by the public"

Kim M. Pepin, Amy J. Davis, Daniel Streicker, Justin W. Fisher, Kurt C. VerCauteren and Amy T. Gilbert

## SM1 Raw Data Information

Table **SM1.1**: Summary of NWRC enhanced surveillance of no exposure (i.e. no high-risk contact) reports. The proportion of enhanced surveillance relative to the total number of surveillance samples is shown in parentheses.

| Report Type | 2012 | 2013 | 2014 | Total |
|---|---|---|---|---|
| Negative | 54 (0) | 35 (0) | 18 (0.39) | 107 (0.07) |
| Positive | 55 (0) | 59 (0.02) | 25 (0.52) | 139 (0.10) |
| Total | 109 (0) | 94 (0.01) | 43 (0.47) | 246 (0.09) |

Table **SM1.2**: Summary of the terrestrial animal cases which were tested for rabies, and had location information, in the study area during 2012-2014. The number of rabies positive samples is shown in parentheses. Cases included in the analysis are shown in boldface type.

| | Year | | | | | | Total | |
|---|---|---|---|---|---|---|---|---|
| | 2012 | | 2013 | | 2014 | | | |
| County | **Skunk** | Other | **Skunk** | Other | **Skunk** | Other | **Skunk** | Other |
| Larimer | **67 (34)** | 42 (3) | **44 (35)** | 77 (7) | **12 (1)** | 40 (0) | **123 (70)** | 159 (10) |
| Weld | **32 (21)** | 37 (2) | **23 (14)** | 63 (5) | **13 (12)** | 44 (0) | **68 (48)** | 144 (7) |
| Boulder | **10 (0)** | 22 (0) | **27 (10)** | 28 (1) | **18 (12)** | 26 (3) | **55 (22)** | 76 (4) |
| Total | **109 (55)** | 101 (5) | **94 (59)** | 168 (13) | **43 (25)** | 110 (3) | **246 (139)** | 379 (21) |

Table **SM1.3**: Summary of rabies positive cases that were sequenced. The proportion of samples relative to the total rabies positive cases for each cell is shown in parentheses.

| County | 2012 | 2013 | 2014 | Total |
|---|---|---|---|---|
| Larimer | 1 (0.03) | 16 (0.46) | 1 (1.0) | 18 (0.26) |
| Weld | 3 (0.14) | 1 (0.07) | 12 (1.0) | 16 (0.34) |
| Boulder | 0 (0) | 1 (0.1) | 10 (0.83) | 11 (0.5) |
| Total | 4 (0.07) | 18 (0.31) | 23 (0.92) | 45 (0.32) |

## SM2 Model Formulation

The model below shows the number of infected animals at grid cell i and time t ($y_{it}$), which is a function of whether or not rabies is present at that site and time ($z_{it}$) and the number of samples taken ($R_{it}$) and the prevalence probability ($p$).

The true presence/absence status ($z_{it}$) is conditionally modeled on the previous time step as a Bernoulli random variable with the probability of $\Psi_{it}$. $\Psi_{it}$ is modeled as a function of the transition rates as follows: the local persistence of rabies from one time step to the next ($\phi$), the initial colonization probability of rabies ($\gamma$), and the re-colonization probability ($\zeta$). We modeled the initial colonization rate as a function of 1) intercept only, 2) distance to the nearest rabies-positive case , the spatial spread in 3) north/south ($N_i$) or 4) east/west ($E_i$) directions, 5) a local neighborhood infection density ($Q_{it}$), 6) a seasonal effect with two levels ($S_t$, and 7) a spatial kernel effect. We quantified the influence of these effects using different model structures shown in the table **SM2.4**. The distance between site i at time t and all infected sites is represented by $d_{ijt}$ We also considered two different specifications for the prevalence parameter $p$ (one including an effect of human populations size ($H$) - see General Model Specification below).

## SM2.1 General Model Specification

$$y_{it} = \begin{cases} 0 & , z_{it} = 0, \qquad i = 1, ..., M \\ Bin(p, R_{it}) & , z_{it} = 1, \qquad t = 1, ..., T \end{cases}$$

$$p \sim Unif(0,1) \quad or \quad logit(p_i) = \beta_{p0} + \beta_{p1} * H$$

$$z_{i1} \sim Bern(\psi_{i1}) \qquad \psi_{i1} \sim Unif(0,1)$$

$$z_{it} | z_{it-1} \sim Bern(\Psi_{it})$$

$$\Psi_{it} = \phi_{it-1} z_{i,t-1} + \gamma_{it-1}(1 - z_{it-1})(1 - A_{it-1}) + \zeta_{it-1}(1 - z_{it-1}) A_{it-1}$$

$$\phi_{it} \sim Beta(\alpha_\phi, \beta_\phi)$$

$$\zeta_{it} \sim Beta(\alpha_\zeta, \beta_\zeta)$$

$$logit(\gamma_{it}) = \boldsymbol{X\beta_\gamma} \qquad \boldsymbol{\beta_\gamma} \sim Norm(\boldsymbol{0}, I)$$

## SM2.2 Models on Initial Colonization

Below is the full joint distribution for the dynamic occupancy model. For ease of implementation we show the full conditional distributions for each parameter below. We used a Markov Chain Monte Carlo (MCMC) to generate posterior distributions for each parameter. Based on the conditional distributions shown below we sampled from the conjugate posterior distribution when possible otherwise we used a Metropolis-Hastings (MH) step. We used a normal proposal distribution for all MH steps using the current value ($\theta^{(k)}$) as the mean for the proposal distribution. We used the variance as the tuning parameter and adjusted that value as necessary to reach convergence.

The prior distributions were selected to be relatively uninformative. We ensured the prior choice was not influencing the posterior distributions by visually verifying that the posterior and prior distributions were not overwhelmingly overlapping. We demonstrated this

Table **SM2.4**: **Model specifications**

| | Model name | Function: $logit(\gamma_{it}) =$ |
|---|---|---|
| 1 | Dot model | ** $\gamma_{it} \sim Beta(\alpha_\gamma, \beta_\gamma)$ ** |
| 2 | Distance | $\beta_0 + \beta_1 * \exp(-\beta_2 * min(d_{ijt}))$ |
| 3 | North*Time | $\beta_0 + \beta_1 * N_i + \beta_2 * T + \beta_3 * N_i * T$ |
| 4 | East*Time | $\beta_0 + \beta_1 * E_i + \beta_2 * T + \beta_3 * E_i * T$ |
| 5 | Neighborhood | $\beta_0 + \beta_1 * Q_{it}$ |
| 6 | Season | $\beta_0 + \beta_1 * Spring$ |
| 7 | Kernel | $\beta_0 + \beta_1 * \sum_{it \in z_{it}=0} \frac{1}{d_{ijt}}$ |
| 8 | Distance + North*Time | $\beta_0 + \beta_1 * \exp(-\beta_2 * d_{it}) + \beta_3 * N_i + \beta_4 * T + \beta_5 * N_i * T$ |
| 9 | Distance + Neighborhood | $\beta_0 + \beta_1 * \exp(-\beta_2 * d_{it}) + \beta_3 * Q_{it}$ |
| 10 | North*Time + Neighborhood | $\beta_0 + \beta_1 * N_i + \beta_2 * T + \beta_3 * N_i * T + \beta_4 * Q_{it}$ |
| 11 | Distance + Season | $\beta_0 + \beta_1 * \exp(-\beta_2 * d_{it}) + \beta_3 * S_t$ |
| 12 | North*Time+Season | $\beta_0 + \beta_1 * N_i + \beta_2 * T + \beta_3 * N_i * T + \beta_4 * S_t$ |
| 13 | Neighborhood + Season | $\beta_0 + \beta_1 * Q_{it} + \beta_2 * S_t$ |
| 14 | Kernel + North*Time | $\beta_0 + \beta_1 * N_i + \beta_2 * T + \beta_3 * N_i * T + \beta_4 * \sum_{it \in z_{it}=0} \frac{1}{d_{ijt}}$ |
| 15 | Kernel + Season | $\beta_0 + \beta_1 * \sum_{it \in z_{it}=0} \frac{1}{d_{ijt}} + \beta_2 * S_t$ |
| 16 | Distance + Neighborhood + Season | $\beta_0 + \beta_1 * \exp(-\beta_2 * d_{it}) + \beta_3 * Q_{it} + \beta_4 * S_t$ |

by showing the posterior and prior distributions for or top predictively model ($\gamma(distance + season) + p(Hpop)$), Figure **SM2.1**. The figure shows an example with 50,000 MCMC iterations, here the burn-in was 10,000 iterations.

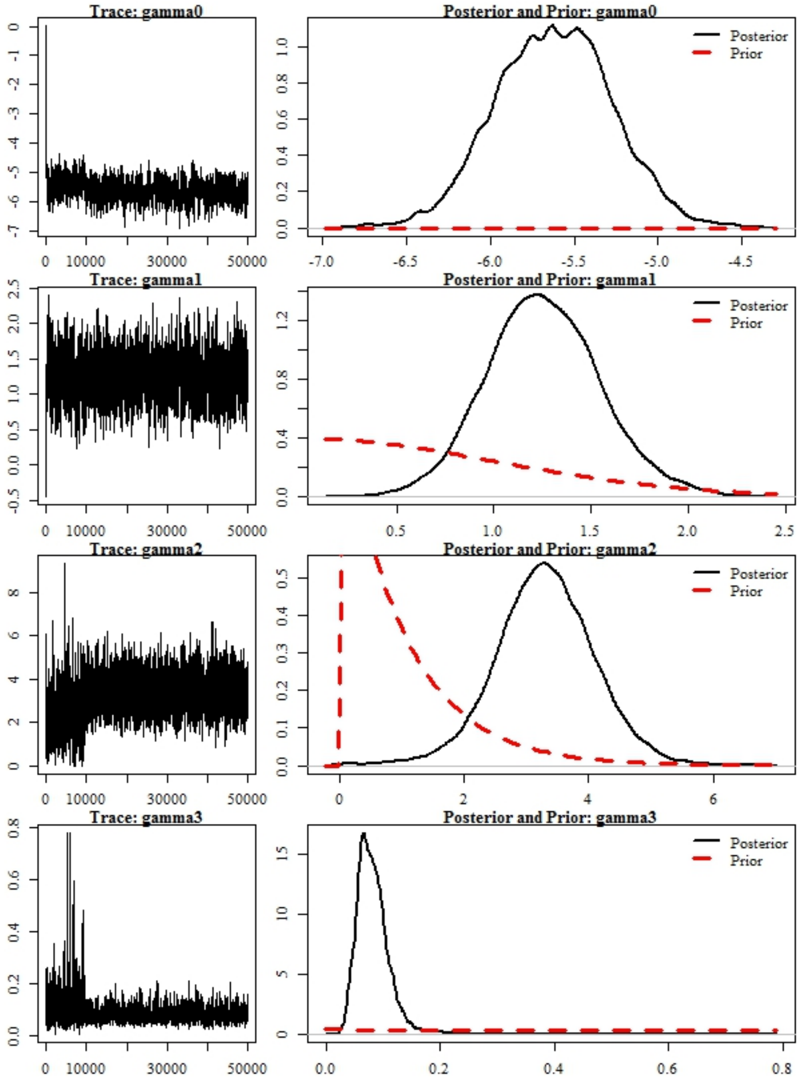


Figure **SM2.1**: **Trace plots and posterior densities**. Trace plots (left column) and posterior densities (right column) shown for the beta parameters from the top predictive model on initial colonization (distance and season). The posterior densities (black line) are shown with the prior distributions (red line) for comparison. Plots like these were used to unsure convergence and mixing for all parameters.

# SM3 Work Flow

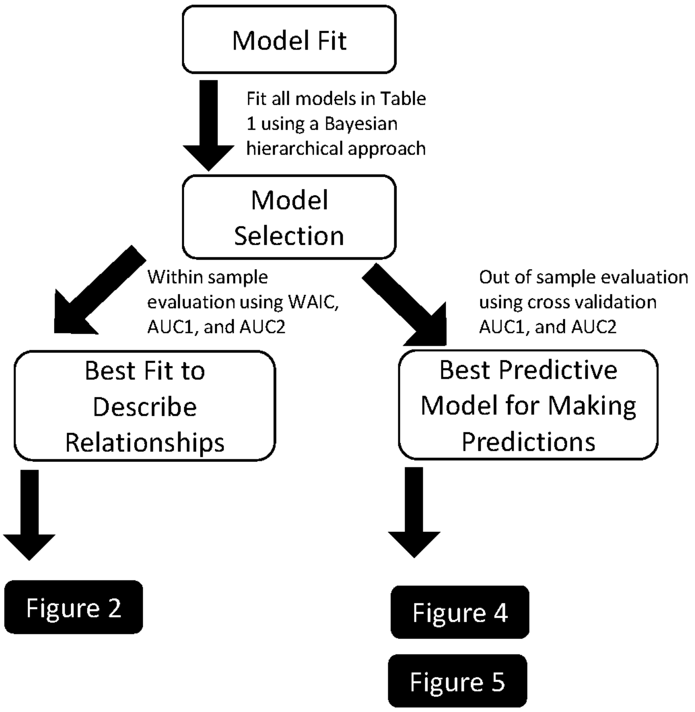


Figure **SM3.1**: Schematic of work flow for model fit, model selection, and model evaluations used in this manuscript.

## SM3.1 Joint Distribution

$$[\boldsymbol{z},\boldsymbol{\psi},\boldsymbol{\phi},\boldsymbol{\gamma},\boldsymbol{\zeta},\boldsymbol{p}|\boldsymbol{y},\boldsymbol{D}] \propto \prod_{i=1}^{R}\left(\prod_{t=1}^{T}[y_{it},J_{it}|p]^{z_{it}}1^{(1-z_{it})}[z_{it}|z_{it-1}]\right)[\psi_1][\phi][\boldsymbol{\beta_\gamma}][\zeta][p]$$

### SM3.1.1 Conditional distributions

$$[\psi_1|\bullet] \propto \prod_{i=1}^{R} [z_{i1}|\psi_{i1}][\psi_{i1}]$$
$$\sim Beta\left(\sum_{i=1}^{R} z_{it} + 1, \sum_{i=1}^{R} (1 - z_{it}) + 1\right)$$
$$[\phi|\bullet] \propto \prod_{i=1}^{R}\prod_{t=1}^{T} [z_{it}|z_{i,t-1}][\phi]$$
$$\sim Beta\left(\sum_{i\in z_{t-1}=1}^{R} z_{it} + 1, \sum_{i\in z_{t-1}=1}^{R} (1 - z_{it}) + 1\right)$$
$$[\boldsymbol{\beta_\gamma}|\bullet] \propto \prod_{i\in z_{t-1}=0, A_{t-1}=0} \left([z_{it} * A_{it}|\gamma_{it}]\right)[\boldsymbol{\beta_\gamma}]$$

Metropolis-Hastings

$$MHratio = \left(\frac{\prod_{i\in z_{t-1}=0, A_{t-1}=0} Bern(x_{it}\boldsymbol{\beta_\gamma}^*|z * A}{\prod_{i\in z_{t-1}=0, A_{t-1}=0} Bern(x_{it}\boldsymbol{\beta_\gamma}|z * A}\right)$$
$$\times \left(\frac{Norm(\boldsymbol{\beta_\gamma}^*, 0, 1)}{Norm(\boldsymbol{\beta_\gamma}, 0, 1)}\right)$$
$$[\zeta|\bullet] \propto \prod_{i=1}^{R}\prod_{t=1}^{T} [z_{it}|z_{i,t-1}][\zeta]$$
$$\sim Beta\left(\sum_{i\in z_{t-1}=0, A_{t-1}=1}^{R} z_{it} + 1, \sum_{i\in z_{t-1}=0, A_{t-1}=1}^{R} (1 - z_{it}) + 1\right)$$
$$[z_{it}|\bullet] \propto \prod_{i\in y=0} \left([y_{it}, J_{it}|p]^{z_{it}} 1^{(1-z_{it})}\right)[z_{it}|z_{it-1}, z_{it+1}]$$
$$\sim Bern\left(\frac{\prod_{i\in y=0}(1-p)\psi^*_{it}}{\prod_{i\in y=0}(1-p)\psi^*_{it} + (1-\psi^*_{it})}\right)$$

$\psi^*$ is dependant on the status of $z_{t-1}$ and $z_{t+1}$ described in the conditions below

$\psi^*_{A, z_{t-1}, z_{t+1}}$

$$[\psi^*_{\cdot,1,1}] = \frac{\phi_{t-1}\phi_t}{\phi_{t-1}\phi_t + (1-\phi_{t-1})\zeta_t}$$
$$[\psi^*_{\cdot,1,0}] = \frac{\phi_{t-1}(1-\phi_t)}{\phi_{t-1}(1-\phi_t) + (1-\phi_{t-1})(1-\zeta_t)}$$

$$[\psi^*_{0,0,1}] = \frac{\gamma_{t-1}\phi_t}{\gamma_{t-1}\phi_t + (1-\gamma_{t-1})\gamma_t}$$
$$[\psi^*_{0,0,0}] = \frac{\gamma_{t-1}(1-\phi_t)}{\gamma_{t-1}(1-\phi_t) + (1-\gamma_{t-1})(1-\gamma_t)}$$
$$[\psi^*_{1,0,1}] = \frac{\zeta_{t-1}\phi_t}{\zeta_{t-1}\phi_t + (1-\zeta_{t-1})\zeta_t}$$
$$[\psi^*_{1,0,0}] = \frac{\zeta_{t-1}(1-\phi_t)}{\zeta_{t-1}(1-\phi_t) + (1-\zeta_{t-1})(1-\zeta_t)}$$

The $\psi^*_{it}$ values are easily manipulated for t=1 and t=T.

## SM4 Model Selection Methods

We examined several different methods to compare model performance of our model set. We started with some typical model selection methods for Bayesian hierarchical models (Watanabe Akaike Information Criteria; WAIC) and assessing model fit for binary data (area under the receiver operator curve; AUC). Since we were particularly interested in the predictive ability of our model we used a series of cross validation metrics. Because our data set was small we used a K-fold cross validation method often called leave-one-out cross validation (Hooten and Hobbs (2015)). This method involves leaving one time step out and estimating the parameters given the rest of the data then predicting the data from the missing time step. There were four months in our study in which there were no samples taken and we could not validate results from these months and felt that predictions before or after two consecutive missing months would be less reliable thus we predicted a total of 23 months. Additionally, we ran the same model selection metrics with the out-of-sample data: WAIC and AUC. The methods are described in detail below.

## SM4.1 WAIC: WATANABE (2013)

$$WAIC = -2\sum_{i=1}^{n} log \int [y_i|\theta][\theta|y]d\theta + 2p_{D,2} \quad (1)$$

$$p_{D,2} = \sum_{i=1}^{n} var_{\theta|y}(log[y_i|\theta]) \quad (2)$$

## SM4.2 AUC 1: FIT FOR PREVALENCE PROCESS (WHICH INCLUDES OBSERVATION PROCESS), FAWCETT (2006)

R Code resulting in the first AUC statistic: $y_{it}$ predictions compared to $y_{it}$ observed

```
### Determine posterior z values (indicator of presence of rabies)
zs=apply(z[,,-(1:n.burn)],c(1,2),mean)
### Create the index values for all z’s that were 1
idx=which(zs==1,arr.ind=TRUE)
### Predict y values, accounting for prevalence only when rabies is present
ypred=matrix(0,nsites,months)
ypred[idx]=rbinom(dim(idx)[1],size = n[idx],prob = mean(p[-(1:n.burn)]))
### Caluclate the AUC for the real y values compared to the predicted y values
auc(c(y),c(ypred))
```

## SM4.3 AUC 2: FIT FOR OCCUPANCY PROCESS, FAWCETT (2006)

R Code resulting in the second AUC statistic: $z_{it}$ observed compared to $\Psi_{it}$ predicted

```
### Determine posterior psi values (occupancy probabilities)
psi.est=apply(psi[,,-(1:n.burn)],c(1,2),function(x)mean(x,na.rm=TRUE))
```

```
### Calculate the z values from the real data (i.e. z=1 if y>0)
zreal=c(ifelse(y>0,1,0))
### Compute the AUC for the estimated psi values compared to the z values
### (ignoring prevalence)
roc(zreal,psi.est)
```

### SM4.4 Cross-validation: Out-of-sample cross validation, Hooten and Hobbs (2015)

$$CV = -2\sum_{k=1}^{K} log\left(\frac{\sum_{l=1}^{L} Binom(y_k|n_k, p^{(l)} z_k^{(l)})}{L}\right) \tag{3}$$

## SM5 Calculation of Transmission Distance and Local Neighborhood Infection Density Impact

For models that included the distance parameter, we calculated a single metric to demonstrate this relationship i.e. the transmission distance. We defined the transmission distance as the distance to the nearest infected neighbor that was half the probability of initial colonization than the probability of initial colonization at zero distance to the nearest infected neighbor (i.e., the maximum). The equations below show the calculation of transmission distance for the model with just the distance decay parameter, which can be modified for any function that includes the distance decay parameter. $\gamma_0$ denotes the initial colonization probability at zero-distance.

$$\gamma_0 = logit^{-1}(\beta_0 + \beta_1) \tag{4}$$

$$transmission - distance = \frac{-log(\frac{logit(\frac{\gamma_0}{2}) - \beta_0}{\beta_1})}{\beta_2} \tag{5}$$

To calculate the impact of local neighborhood infection density ($Q_{it}$) in more explicit terms we examined the average impact of one additional infected neighbor on initial colonization probability ($\gamma$). At most there could be eight infected neighbors for any grid cell. Therefore, we looked at the initial colonization probability given the posterior mean values for the parameter effects in the top model (betas) as the $Q_{it}$ value changed from 0 to 8 of 8. Due to the logit link function the relationship is not directly linear and thus we took the mean of the one neighbor increases to calculate the impact of a one neighbor increase on initial colonization. To calculate the credible intervals we used the lower and upper 95% credible intervals for the beta associated with the infection density term.

## SM6 Model Validation Method

To ensure our model was able to estimate model parameters correctly, we simulated data using the model above under a range of parameter values and estimated the parameter values that were input. We then compared the estimated parameters to those from which the data were simulated (i.e., the "truth") to verify that parameter values could be recovered at least 95% of the time. We focused on the model's ability to recover the beta values for the effects on initial colonization ($\gamma$) as model complexity increased. For the non-linear effects (distance and space by time interaction) we tested whether we could recover the true transmission distance value (defined above) from simulated data and the decay curve. An example of the relationship between the estimated distance curve relative to the true value from simulated data is shown in Figure **SM6.1**. For the space by time relationships we looked at the

average space by time values comparing the simulated values to the estimated parameters (example in Figure **SM6.2**). For the local neighborhood infection density we compared the estimated values to the simulated data to ensure the general pattern held true (example in Figure **SM6.3**).

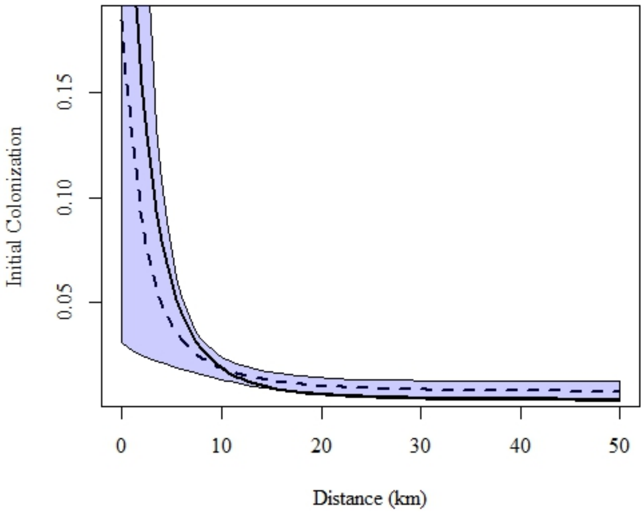


Figure **SM6.1**: **Posterior distance validation**. Initial colonization probability as a function of distance to nearest infected neighbor for true values (solid line, generated from simulated data) and estimates from the simulated data (dashed with shaded 95% credible interval).

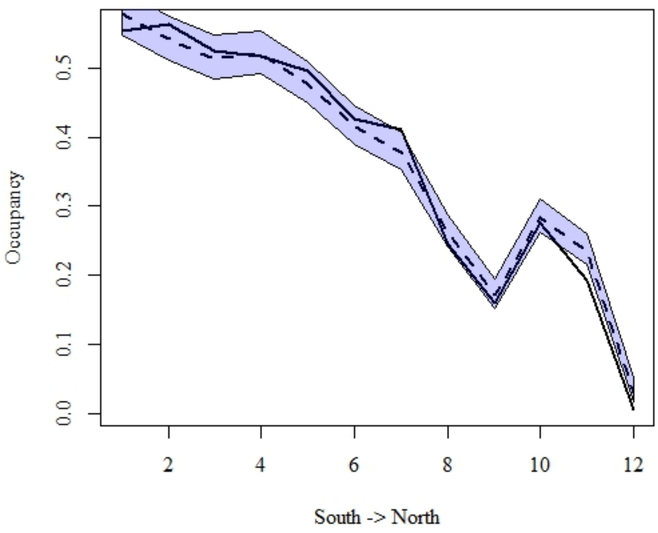


Figure **SM6.2**: **Posterior direction validation**. Average occupancy probability across the north-south gradient based on the simulated data (solid line) and estimates from the simulated data (dashed line with shaded 95% credible interval).

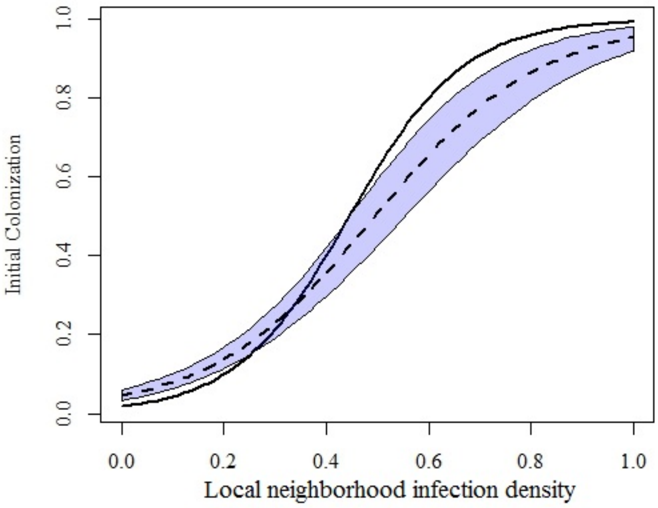


Figure **SM6.3**: **Posterior density validation**. Initial colonization probability as function of local neighborhood infection density for true values (solid lines, generated from simulated data) and estimates from the simulated data (dashed line with shaded 95% credible interval.

# References


Fawcett, T. (2006). An introduction to roc analysis. *Pattern recognition letters 27*(8), 861–874.

Hooten, M. and N. Hobbs (2015). A guide to bayesian model selection for ecologists. *Ecological Monographs 85*(1), 3–28.

Watanabe, S. (2013). A widely applicable bayesian information criterion. *The Journal of Machine Learning Research 14*(1), 867–897.
