# Supplementary material for: Predicting spatial spread of rabies in skunk populations using surveillance data reported by the public
Source: PLoS Negl Trop Dis. 2017 Jul 31;11(7):e0005822. doi: 10.1371/journal.pntd.0005822 (PMC5552346; doi:10.1371/journal.pntd.0005822)
Supplement: S1 Figures — Additional figures showing parameter fits and spatial spread. (PDF) [file pntd.0005822.s002.pdf]

Supporting Information 1: Figures of results "Predicting spatial spread of rabies in wildlife populations using surveillance data reported by the public"

Kim M. Pepin, Amy J. Davis, Daniel Streicker, Justin W. Fisher, Kurt C. VerCauteren and Amy T. Gilbert

SR1 Results from Directional Models

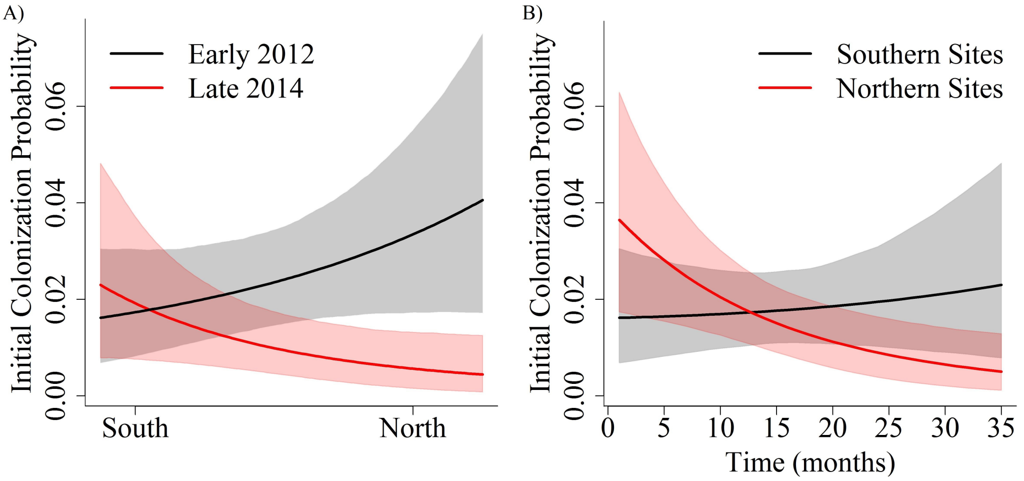

Figure **SR1.1**: **North/South by time**. A) Initial colonization probability based on a north to south gradient at the beginning of our study (Jan 2012) and the end of the study (Dec 2014). B) Initial colonization probability across time for northern sites compared to southern sites. 95% credible intervals are shown as shaded regions. These plots were produced using model 4.

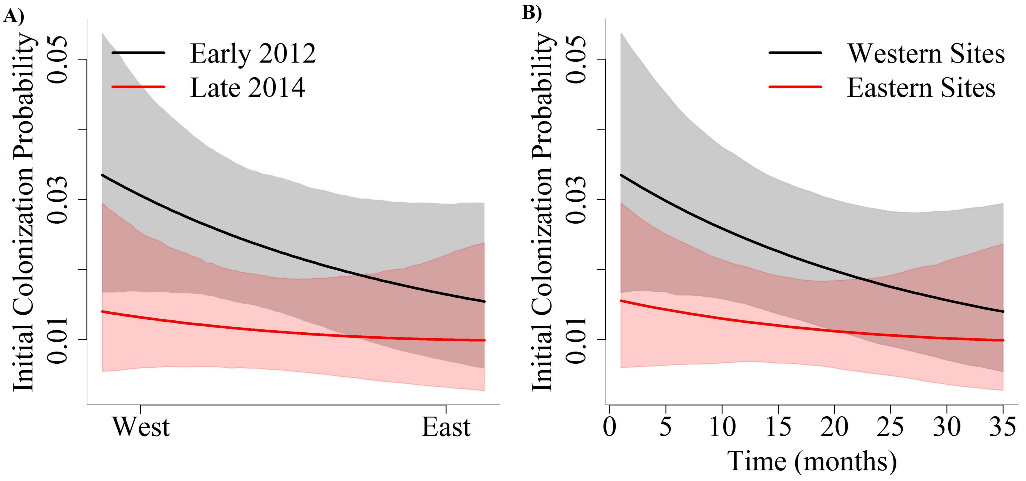

Figure **SR1.2**: **East/West by time**. A) Initial colonization probability based on a west to east gradient at the beginning of our study (Jan 2012) and the end of the study (Dec 2014). B) Initial colonization probability across time for eastern sites compared to western sites. 95% credible intervals are shown as shaded regions. These plots were produced using model 5.

SR2 Out-of-Sample Predictions

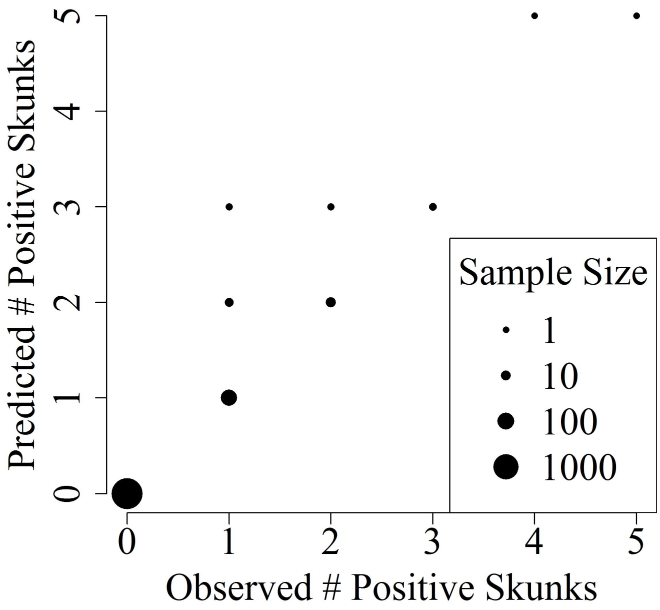

Figure **SR2.1**: **Out-of-sample prediction**. Out of sample prediction from the best predictive model (Table 1, Model 11b). The X-axis shows the predicted number of RABV-positive skunks per grid cell and month against the corresponding observed values. Predicted values were generated completely out-of-sample using only the initial conditions, parameter estimates from the full model and numbers of samples collected. Spearman's correlation coefficient is 0.99 when zeros are included and 0.58 when they are excluded.

SR3 Full Occupancy Results

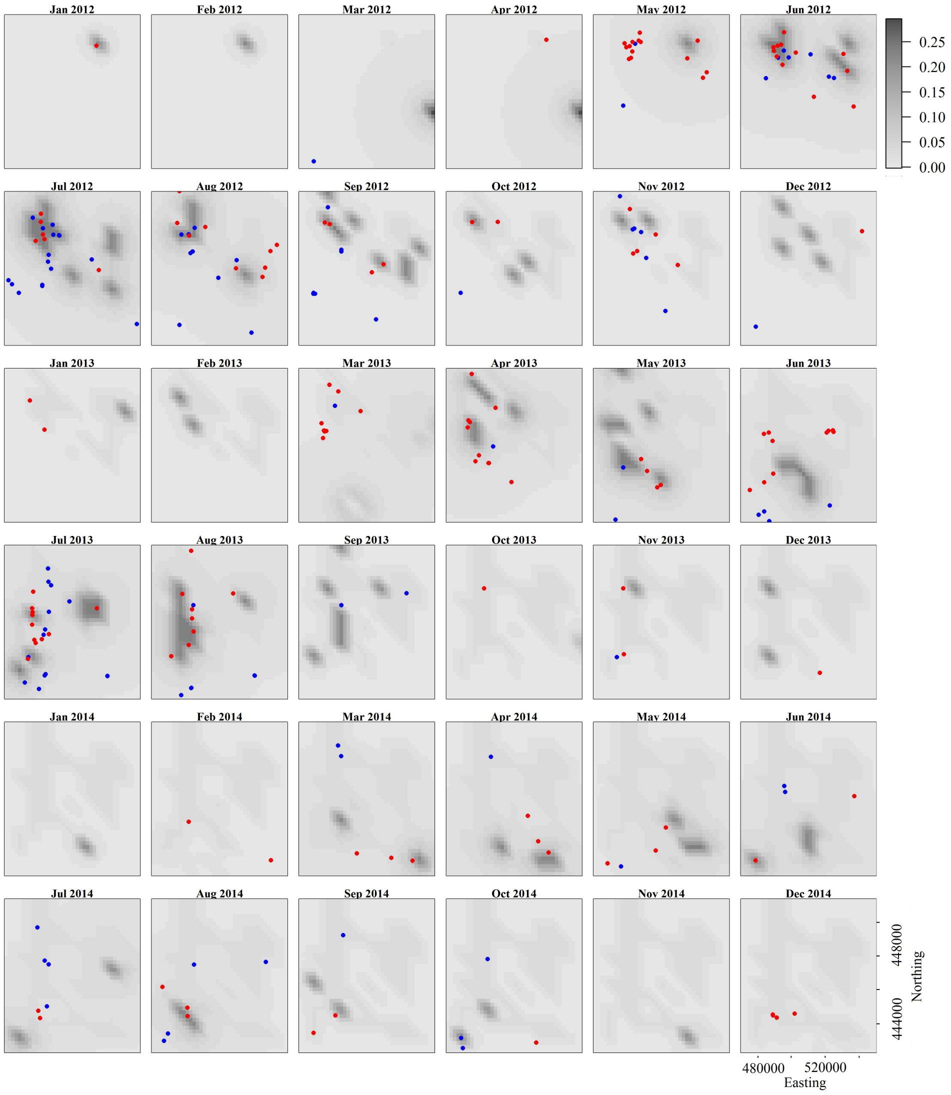

Figure **SR3.1**: **Occupancy dynamics for both clades**. Occupancy probability in space and time from the model with distance and season on initial colonization and human population on prevalence. Each panel represents 1 month (January 2012-December 2014). Darker values represent higher occupancy probability. The location of positive (red) and negative (blue) samples are shown by month. Each plot covers the same area as from Fig. 1 in the main text.

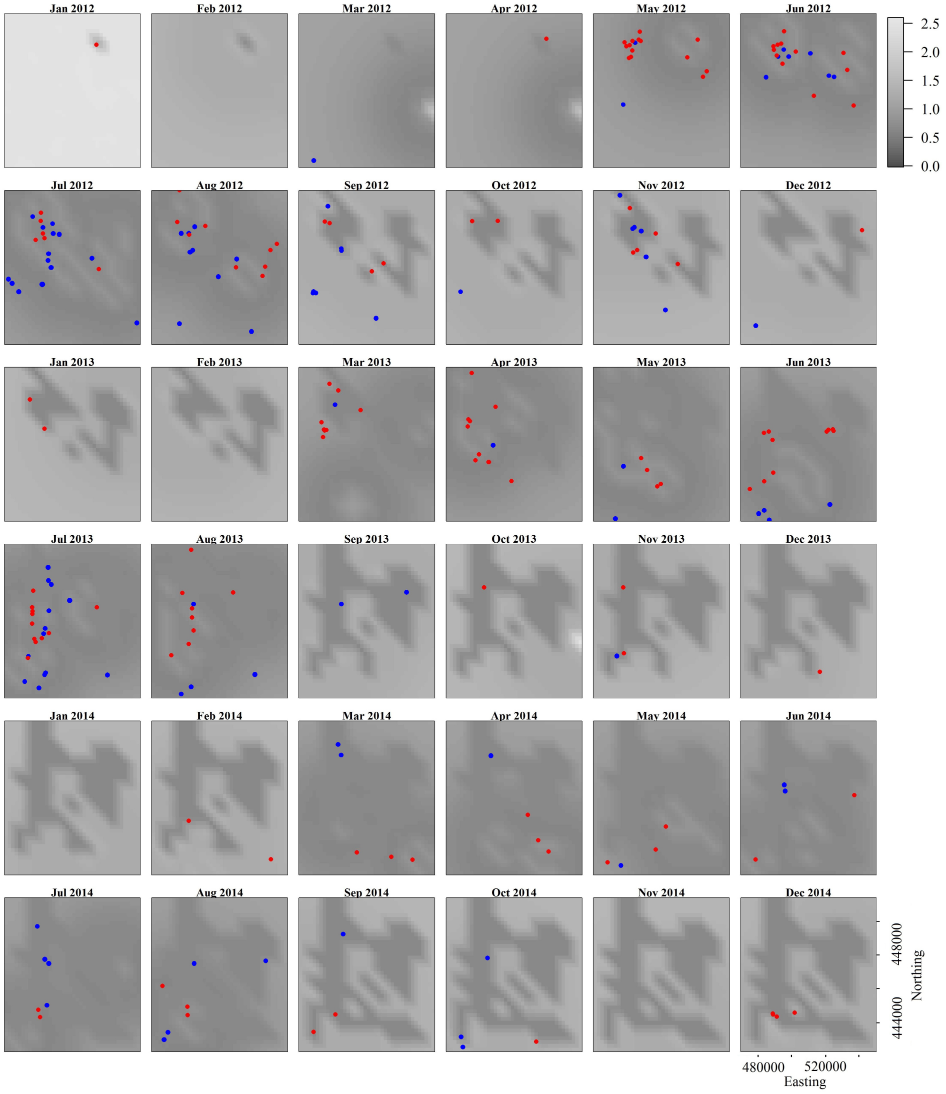

Figure **SR3.2**: **Coefficient of variation for both clades**. Coefficient of variation for occupancy probability in space and time from the model with distance and season on initial colonization and human population on prevalence. Each panel represents 1 month (January 2012-December 2014). Darker values represent higher occupancy probability. The location of positive (red) and negative (blue) samples are shown by month. Each plot covers the same area as from Fig. 1 in the main text, the white points represent the locations of negative samples and the black dots represent locations of positive samples.
